# Unidirectional exceptional point of reflectionless states in a magnonic mirror array

**DOI:** 10.1126/sciadv.aea6000

**Published:** 2026-03-04

**Authors:** Zi-Qi Wang, Yuan-Peng Peng, Yi-Pu Wang, J. Q. You

**Affiliations:** ^1^ Zhejiang Key Laboratory of Micro-Nano Quantum Chips and Quantum Control, School of Physics, and State Key Laboratory for Extreme Photonics and Instrumentation, Zhejiang University, Hangzhou 310027, China.; ^2^ College of Optical Science and Engineering, Zhejiang University, Hangzhou 310027, China.

## Abstract

Exceptional points (EPs) in non-Hermitian systems are singularities where both eigenvalues and eigenvectors coalesce. In scattering systems, EPs correspond to the merging of scattering states, leading to reflectionless (RL) behavior. A reflectionless exceptional point (RL EP) arises when two RL states further coalesce, yielding an anomalous quartic spectral response. While RL EPs have been explored in bidirectional systems, their unidirectional realization remains elusive. Here, we experimentally demonstrate a unidirectional RL EP by engineering collective states in an anti-Bragg magnonic mirror array. Inversion symmetry is broken using a giant spin ensemble that couples to a waveguide at three spatially separated points, enabling unidirectional RL. At the RL EP, the reflection spectrum flattens and broadens substantially beyond the Lorentzian profile. The observed spectral valleys also expose dark state behaviors that are typically inaccessible through conventional measurements. Our results provide a route toward controlling collective coherence in open systems and developing broadband unidirectional devices.

## Teaser

A unidirectional reflectionless magnonic mirror array widens the silence of reflection into a broad band at the exceptional point.

## INTRODUCTION

Exceptional points (EPs) in non-Hermitian systems correspond to degeneracies where both eigenvalues and eigenvectors coalesce, often giving rise to anomalous wave responses (*1*–*5*). In scattering systems, scattering EPs are typically associated with the formation of reflectionless (RL) states, where coalescence of scattering eigenchannels yields zero reflection at a particular frequency. Unidirectional RL states, in which reflection vanishes in one direction but remains strong in the opposite, are especially appealing for applications in unidirectional invisibility. This concept has been extensively investigated in parity-time (PT) symmetric optical structures (*1*–*4*, *6*–*9*) and can be adapted to realize asymmetric reflection between distinct polarization states in non-Hermitian metasurfaces (*10*–*12*). Nevertheless, the unidirectional RL states induced by EPs in previous studies are generally narrowband (*2*, *6*, *10*–*17*) limited by the Lorentzian profile of isolated resonances. Recently, a special type of EP, known as the RL EP, has been proposed to enhance spectral performance (*18*–*22*). At an RL EP, two degenerate RL solutions merge, giving rise to a quartic spectral response that notably broadens the RL bandwidth without requiring external gain or complex control parameters (*17*, *23*–*29*). Up to now, all RL EPs demonstrated have been confined to symmetric or bidirectional platforms. The emergence of unidirectional behavior requires an additional ingredient involving the breaking of spatial symmetry such as inversion or mirror symmetry in the system’s geometry or dissipation. Thus, experimentally achieving and controlling the necessary asymmetry within a fabricated sample remains challenging.

A radiative emitter array coupled to the waveguide serves as a promising experimental platform for studying these unusual scattering phenomena (*30*–*35*). Each emitter in such a system can act as a mirror at resonance, and its effective reflectivity is determined by its radiation efficiency. Notably, using a giant emitter beyond the dipole approximation allows for flexible control of reflectivity, as its radiative damping can be modulated through the resonance frequency (*36*–*47*). Moreover, the addition of coupling points can further augment this capability, as the collective interference in the giant emitter can considerably enhance its radiation efficiency (*45*). The unique tunability of the giant emitter allows for both the generation and precise adjustment of system asymmetry, facilitating the emergence of distinctive collective effects. In contrast to the conventional setup focusing on isolated resonances in EP-related studies (*48*–*50*), the collective states arising from the collective interactions among these emitters provide an alternative platform for exploring non-Hermitian physics. These interference-enabled collective states, such as long-lived dark states formed via destructive interference, enable rich applications ranging from quantum memories to strong couplings in open environments (*33*, *35*, *40*, *51*–*54*). By engineering the radiative properties of individual emitters, these collective states can be manipulated to achieve a broad range of unconventional wave phenomena.

In this work, we demonstrate that the combination of an RL EP and deliberate inversion-symmetry breaking in a collective magnonic system leads to unidirectional RL behavior with enhanced bandwidth. Leveraging the flexible tunability of the magnonic system (*21*, *55*–*82*), we design an inhomogeneous anti-Bragg magnon array composed of three spatially separated yttrium iron garnet (YIG) spheres coupled to a meandering waveguide. The Kittel mode of magnons (uniform spin precession mode) in each YIG sphere is coupled to the traveling photon mode in the waveguide. The system forms an effective magnonic mirror array (MMA), where two boundary magnonic mirrors create a dark state cavity that coherently couples to the central magnon mode. To break the system’s inversion symmetry, we implement a giant spin ensemble (GSE) scheme (*35*) in which one of the magnonic mirrors is strongly coupled to the waveguide via three spatially separated points, leading to constructively enhanced radiative decay. In contrast, the other two YIG spheres couple to the waveguide at a single point each and can be regarded as two small spin ensembles. This asymmetric spatial configuration readily breaks the central inversion symmetry of the system.

In experiment, we observe that only one reflection direction exhibits the hallmark features of an RL EP, including reflection suppression with a flattened quartic line shape and notably broadened bandwidth. The other direction remains highly reflective, confirming the unidirectional nature of the system. Moreover, by tuning the magnon frequencies, we continuously track the coalescence and splitting of RL states, revealing their coherent origin and strong sensitivity to collective dynamics. Our results showcase a promising route for engineering non-Hermitian phenomena in collective systems and open the door to broadband, unidirectional wave control in both classical and quantum regimes.

## RESULTS

### System and concepts

**Fig. 1.**
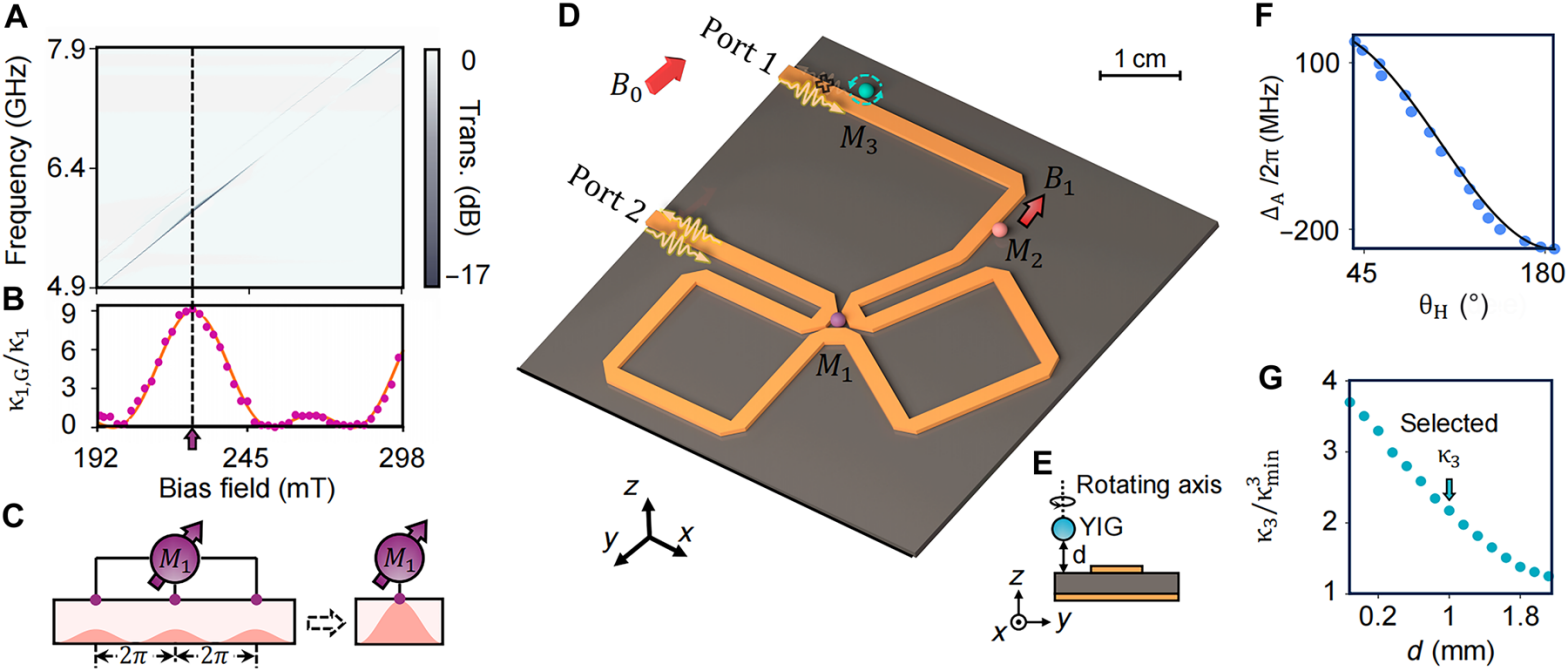
Design of the asymmetric magnon mode array with GSE. (**A**) Transmission mapping of $M_{1}$ as a function of the bias magnetic field, showing periodic modulation in dip depth due to phase-dependent coupling. (**B**) Extracted radiative damping rates of the GSE (dots) as a function of frequency, fitted using Eq. 1 (solid curve). (**C**) Schematic of a GSE with three spatially separated coupling points, enabling collective constructive interference that significantly enhances the radiative coupling between magnon mode $M_{1}$ and the waveguide. (**D**) Experimental setup: YIG spheres $M_{1}$ (purple) and $M_{2}$ (pink) are mounted on the waveguide (orange). The frequency of $M_{2}$ is tuned via a local field $B_{1}$ generated by a coil beneath it. Sphere $M_{3}$ (cyan) is attached to a cantilever connected to a rotation stage. All spheres are magnetized by a global field $B_{0}$. (**E**) Side view showing the adjustable position of $M_{3}$ along the *x* and *z* axes. (**F**) Tunability of $M_{3}$’s resonance frequency via anisotropy field control, plotted versus rotation angle $\theta_{H}$. (**G**) Extracted radiative damping rate $\kappa_{3}$ of $M_{3}$ versus its vertical distance *d* from the waveguide.

Our MMA device is schematically illustrated in Fig. 1D. It consists of a meandering waveguide and three 1-mm-diameter single-crystal YIG spheres. The considered Kittel mode in each sphere is denoted as $M_{i}$, i=1,2,3, which has a total decay rate $\gamma_{i}=\kappa_{i}+\beta$, where $\kappa_{i}$ and β are the radiative and intrinsic losses, respectively. We apply a uniform bias magnetic field along the *y* axis to ensure homogeneous magnetization of each YIG sphere. Once magnetization is saturated, the magnon resonance frequency is given by $\omega_{m}=\gamma \left(H_{e}+H_{A}\right)$, where γ/2π=28 GHz/T is the gyromagnetic ratio, $H_{e}$ is the bias field, and $H_{A}$ denotes the anisotropy field. Notably, magnon mode 1 ($M_{1}$, purple) is coupled to the waveguide at three well-separated mitered corners that are equidistant, resulting in identical local interaction strengths $g_{1}$ between the magnon mode and the waveguide at each point. Along the waveguide, the separation between these coupling points exceeds the microwave wavelength, so the dipole approximation no longer applies. Consequently, interference among the coupling points becomes pronounced and regulates the properties of the GSE. By designing the coupling point separation *L* to be equal, the effective radiative damping rate of the GSE, $\kappa_{1,G}$, can be expressed as (see the Supplementary Materials for details)

$$\kappa_{1,G}=\kappa_{1}\left(3+4\text{cos}\varphi_{G}+2\text{cos}2\varphi_{G}\right)$$ (1)

where $\varphi_{G}=\omega_{1}L/v$ is the accumulated phase between adjacent coupling points. This effective radiative damping rate $\kappa_{1,G}$ can be continuously tuned by varying the magnon resonance frequency, as demonstrated by the dip along the diagonal of the measured transmission spectra versus bias magnetic field shown in Fig. 1A. The depth and width of the transmission dip along the diagonal changes periodically with the resonance frequency, indicating modulation of the magnon-waveguide coupling. These variations are quantitatively extracted by fitting $\kappa_{1,G}$, as presented in Fig. 1B. This observation confirms the successful realization of a GSE (*35*). When the latter two cosine terms in Eq. 1 simultaneously reach their maxima, the magnon-waveguide interactions at the three coupling points of $M_{1}$ interfere constructively, leading to a ninefold enhancement in $\kappa_{1,G}$ relative to the single coupling point value $\kappa_{1}$. In this configuration, the GSE can be simplified as a single coupling point emitter with enhanced and tunable radiative decay, as illustrated in Fig. 1C.

**Fig. 2.**
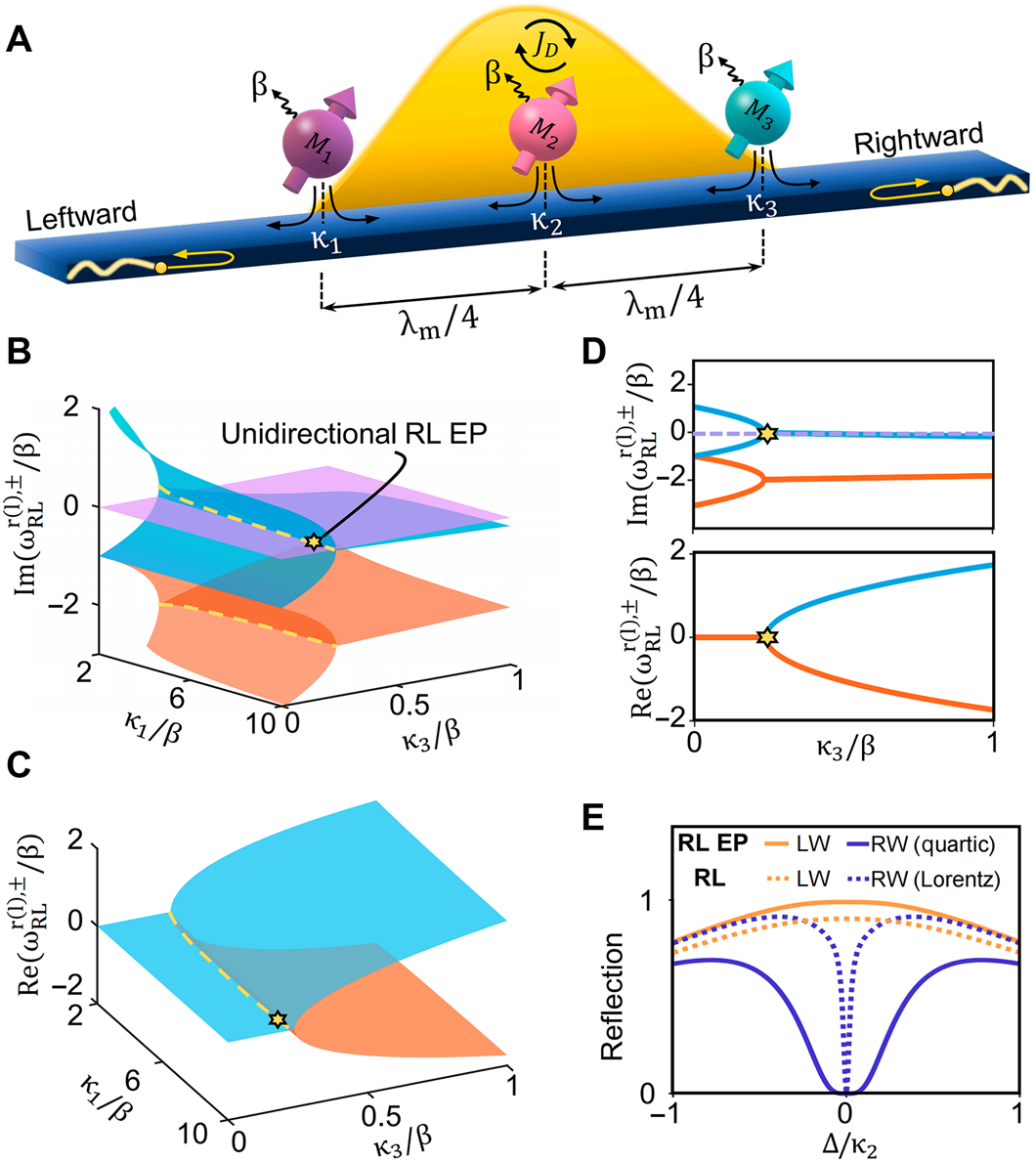
Unidirectional EP of RL states. (**A**) Schematic of the MMA, where three magnon modes are coupled to a waveguide with external (intrinsic) dissipation rates $\kappa_{i}$ (β). Adjacent magnon modes are spaced by $\lambda_{m}/4$. (**B** and **C**) Imaginary (B) and real (C) parts of the RL eigenfrequencies for rightward and leftward incidence, plotted as functions of the magnon radiative decay rates $\kappa_{1}$ and $\kappa_{3}$. Yellow dashed lines indicate the EP conditions. Only the imaginary parts of the RL eigenfrequencies for rightward incidence intersects the zero imaginary frequency (purple) plane, marking the unidirectional RL EP. Here, $\kappa_{2}/\beta =0.93$. (**D**) Real and imaginary parts of the RL eigenfrequencies versus $\kappa_{3}$, extracted from side views of (B) and (C). (**E**) Reflection spectra for both incidence directions under conventional RL (dashed) and RL EP (solid) conditions. Orange and blue curves correspond to leftward and rightward incidence, respectively.

The introduction of the GSE notably enhances the asymmetry of the system, facilitating the subsequent observation of the unidirectional RL states. The realization of these RL states relies on the collective behaviors of the MMA. In this work, we focus on the anti-Bragg condition, where the adjacent magnon modes are spaced by a distance of $\lambda_{m}/4$ (Fig. 2A). Here, $\lambda_{m}/2\pi =v/\omega_{m}$ is the wavelength of the propagating field at the magnon resonance frequency $\omega_{m}$, and *v* denotes the photon group velocity. Under low excitation conditions, the effective Hamiltonian describing this magnonic array is given by

$$H_{\text{eff}}/\hbar =\left(\begin{array}{ccc} \omega_{m}-i\gamma_{1} & J_{12} & i\Gamma_{13}\\ J_{12} & \omega_{m}-i\gamma_{2} & J_{23}\\ i\Gamma_{13} & J_{23} & \omega_{m}-i\gamma_{3} \end{array}\right)$$ (2)

where $J_{\mathrm{sp}}=\sqrt{\kappa_{s}\kappa_{p}}$ and $\Gamma_{\mathrm{sp}}=\sqrt{\kappa_{s}\kappa_{p}}$ (s,p=1,2,3, s≠p) represent the waveguide-mediated coherent and dissipative coupling strengths, respectively, between magnon modes *s* and *p*. To characterize the system’s collective response through spectroscopic reflection measurements, we derive expressions for the rightward and leftward reflection coefficients (see the Supplementary Materials for details)

$$R^{r\left(l\right)}=\frac{{\left(\Delta +\mathrm{i\beta}\right)}^{2}\left(\kappa_{1}-\kappa_{2}+\kappa_{3}\right)-4\kappa_{1}\kappa_{2}\kappa_{3}\pm \delta }{\text{det}\left(\omega \hat{I}-H_{\text{eff}}\right)}$$ (3)

where $\delta =2i\kappa_{2}\left(\Delta +\mathrm{i\beta}\right)\left(\kappa_{1}-\kappa_{3}\right)$, $\Delta =\omega -\omega_{m}$ is the detuning between the probe field and magnon mode, ω is the probe frequency, and $\hat{I}$ is the identity matrix. The key to realizing asymmetric reflection lies in the discriminant term δ, which is only nonzero when the radiative damping rates of the boundary magnon modes are unequal, indicating that the system no longer preserves inversion symmetry. This reflection asymmetry becomes pronounced when the difference between $\kappa_{1}$ and $\kappa_{3}$ is appreciable. The implementation of the GSE readily fulfills this requirement.

To realize the RL state, the numerator of Eq. 3 must vanish, yielding two complex-valued solutions, $\omega_{\text{RL}}^{r,\pm }$ and $\omega_{\text{RL}}^{l,\pm }$, corresponding to the RL conditions for rightward and leftward incidence, respectively. Fixing the radiative decay rate $\kappa_{2}$ of the central magnon mode $M_{2}$, we map the imaginary and real components of $\omega_{\text{RL}}^{r\left(l\right),\pm }$ as functions of $\kappa_{1}$ and $\kappa_{3}$, presented as three-dimensional surfaces in Fig. 2 (B and C). The degeneracy of $\text{Re}\left(\omega_{\text{RL}}^{r,\pm }\right)$ and $\text{Re}\left(\omega_{\text{RL}}^{l,\pm }\right)$ in Fig. 2C results in two distinguishable Riemann sheets. Along the $\kappa_{3}$ axis, these sheets can coalesce further. Despite this spectral degeneracy, the imaginary parts $\text{Im}\left(\omega_{\text{RL}}^{r\left(l\right),\pm }\right)$ differ markedly due to the asymmetry in magnon damping rates across the system. The yellow dashed lines on the surfaces in Fig. 2 (B and C) indicate the EP line of both directions. For these RL states to be experimentally observable, the corresponding solution should be real, i.e., $\text{Im}\left(\omega_{\text{RL}}^{r\left(l\right),\pm }\right)=0$. To identify such conditions, we introduce a zero plane (purple surface) in Fig. 2B to locate intersections with the Riemann surfaces. Notably, only when $\kappa_{1}$ is sufficiently large (e.g., $\kappa_{1}/\beta \approx 10$) can the rightward solutions alone intersect this plane, indicating the emergence of unidirectional RL EP (yellow star) in our system. The side views in Fig. 2D further confirm the presence of unidirectional RL EP. It should be noted that the frequencies $\omega_{\text{RL}}^{r\left(l\right)}$ also correspond to the eigenvalues of the RL Hamiltonian $H_{\text{RL}}^{r\left(l\right)}$ (*16*–*18*, *21*, *22*). The emergence of a unidirectional RL EP in our system essentially stems from $H_{\text{RL}}^{r}$ being PT symmetric, while $H_{\text{RL}}^{l}$ is not (see the Supplementary Materials for details). We compare the corresponding reflection spectra at this RL EP with those of a conventional RL state. As shown in Fig. 2E, the quartic flattening of the reflection profile at the RL EP leads to a broadened unidirectional bandwidth. Note that the parameters used in Fig. 2 (B and C) can be experimentally obtained by individually fitting the transmission response of each magnon mode. Moreover, they can be actively tuned in our fabricated device, as discussed below.

### Realization of the asymmetric MMA via a GSE

**Fig. 3.**
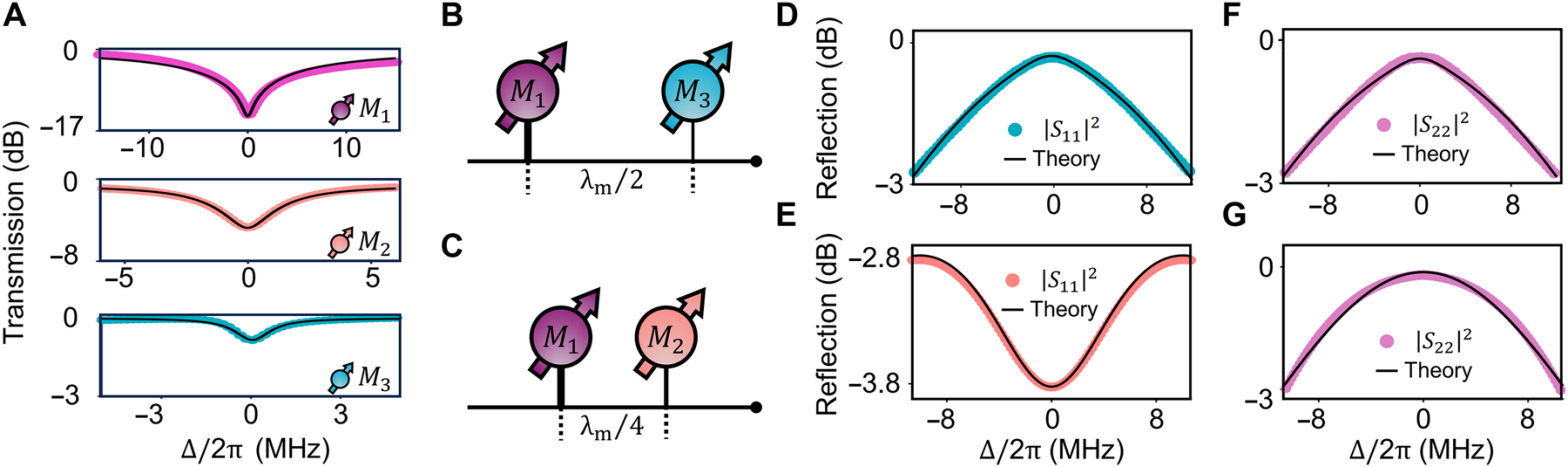
Characterization of relative phases between magnon modes via reflection measurement. (**A**) Individually measured transmission spectra of the three magnon modes (dots) at the GSE resonance frequency, with theoretical fits (black curves). (**B** and **C**) Schematic illustrations of the relative phase configurations: (B) between $M_{1}$ and $M_{3}$, showing dissipative coupling with a π phase difference; (C) between $M_{1}$ and $M_{2}$, showing coherent coupling with a π/2 phase difference. (**D** and **F**) Reflection spectra measured from port 1 (D) and port 2 (F) when only $M_{1}$ and $M_{3}$ are present and dissipatively coupled. (**E** and **G**) Reflection spectra measured from port 1 (E) and port 2 (G) when only $M_{1}$ and $M_{2}$ are present and coherently coupled. All measurements were performed with only the corresponding pair of magnon modes present in the system.

A strongly enhanced radiative coupling in one of the magnonic mirror modes serves as a necessary precondition for realizing the unidirectional RL EP. However, this remains a major challenge to achieve in conventional magnonic systems (*21*, *35*, *56*–*59*, *69*, *83*). This limitation underscores the crucial role of the GSE introduced in our work, which offers a method for fulfilling this condition. At the constructive interference frequency ($\omega_{1}/2\pi =5.9$ GHz), the substantially enhanced radiative coupling produces a broad Lorentzian transmission spectrum, as shown in the top of Fig. 3A. The fitted radiative damping rate of $M_{1}$ is $\kappa_{1,G}/2\pi =13.23$ MHz. The individually measured transmission spectra for the other configured two magnon modes, $M_{2}$ (pink) and $M_{3}$ (green), are also shown in Fig. 3A, with fitted radiative damping rates $\kappa_{2}/2\pi =0.76$ MHz and $\kappa_{3}/2\pi =0.19$ MHz, respectively. The intrinsic damping rates of all the three modes are fitted to β/2π=1.02 MHz. The reduction of $\kappa_{3}$ is clearly identified by a shallower resonance dip, which is controlled by displacing the corresponding sphere away from the microstrips (Fig. 1G; see Materials and Methods for details). This MMA configuration meets the requirement for RL EP formation. Next, we adjust the relative positions of these spheres so that they can meet the anti-Bragg condition.

To explore the collective behaviors of the three magnon modes, their frequencies should be tuned into mutual resonance. For $M_{2}$, this is achieved by applying a local magnetic field $H_{1}$ via a coil beneath the sample. For $M_{3}$, its resonance frequency can also be individually regulated by harnessing the magnetocrystalline anisotropy field (Fig. 1F; see Materials and Methods for details). This versatile tunability enables the realization and comprehensive characterization of the MMA system and its RL EPs.

As the magnon modes are collectively coupled to the waveguide, traveling photons mediate long-range interactions among them. To satisfy the anti-Bragg condition for the magnon array at the GSE resonance frequency $\omega_{1}$, the accumulated propagation phase $\varphi_{\text{mn}}$ between the magnon modes $M_{m}$ and $M_{n}$ should be precisely controlled by adjusting the spacing between the corresponding YIG spheres via step motors. In our implementation, magnon modes $M_{2}$ and $M_{3}$ are individually coupled to the GSE magnon mode $M_{1}$ at 5.9 GHz. To ensure the stable emergence of the RL EP, the magnon resonance frequency is chosen to avoid perturbations from higher-order magnetostatic modes, and the input power is −20 dBm to suppress Kerr nonlinearity–induced frequency shifts.

**Fig. 4.**
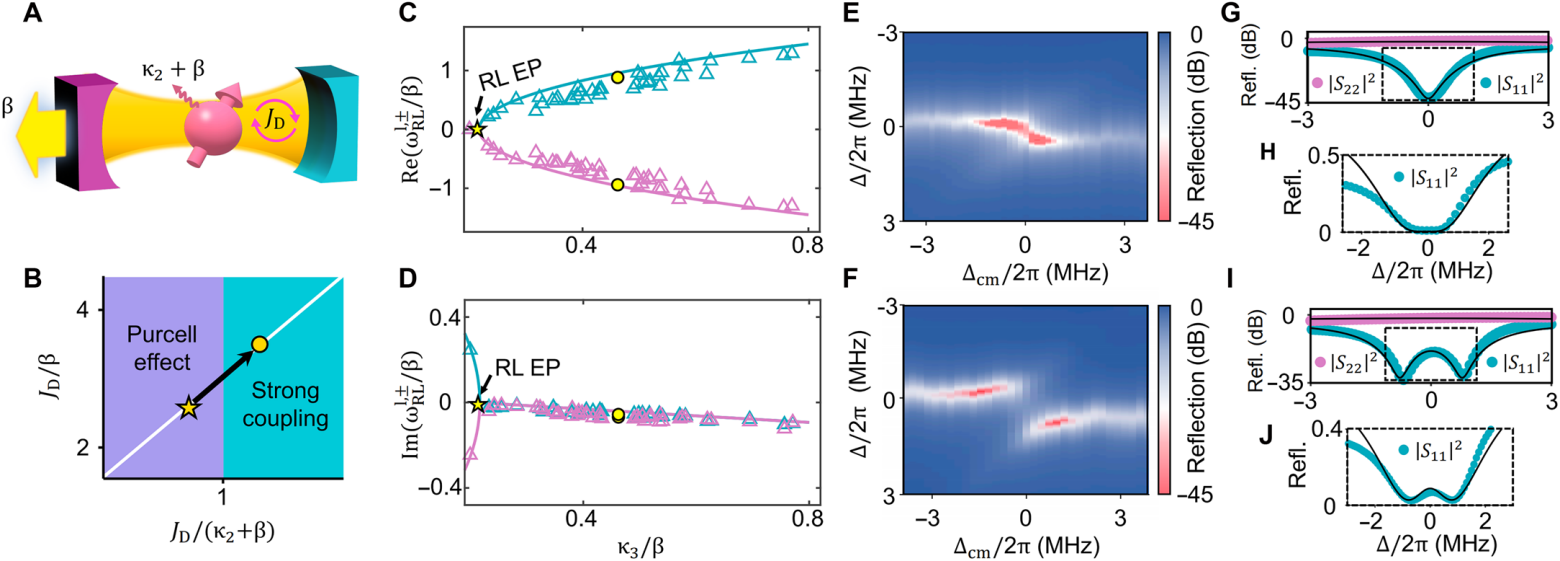
Unidirectional detection in the effective magnonic mirror cavity system. (**A**) Schematic illustration of the effective magnonic mirror cavity system, where a probing magnon mode couples to a dark cavity mode formed by two dissipatively coupled magnonic mirrors. (**B**) Parameter space showing two coupling regimes, separated by the ratio between the coupling strength $J_{D}$ and the linewidths of the cavity and magnon modes. The white line tracks the system’s trajectory as $\kappa_{3}$ is tuned. Stars and circles mark the cases in (E) and (F). (**C** and **D**) Fitted real (C) and imaginary (D) parts of the RL-state eigenfrequencies $\omega_{\text{RL}}^{l,\pm }$ versus $\kappa_{3}/\beta$. (**E** and **F**) Reflection mappings of ${|S_{11}|}^{2}$ measured by gradually rotating sphere $M_{3}$ in two different coupling regimes: $\kappa_{3}/2\pi =0.19$ MHz (E) and 0.55 MHz (F). (**G** and **I**) Reflection spectra measured at resonance. Cyan and purple dots represent ${|S_{11}|}^{2}$ and ${|S_{22}|}^{2}$ extracted from (E) and (F), respectively. Black curves show theoretical fits. (**H** and **J**) The ${|S_{11}|}^{2}$ reflection spectra in (G) and (I) plotted on a linear scale.

As illustrated in Fig. 3B, the magnon modes $M_{1}$ and $M_{3}$, serving as the magnonic mirrors of the MMA, are separated by a π phase shift, forming a dissipatively coupled configuration. Despite the strong inversion asymmetry between these two mirrors, the reflection spectra in both directions, shown in Fig. 3 (D and F), appear nearly identical. This arises because only the bright eigenmode is accessible in the reflection measurements, producing a broad, high-reflectivity peak in both directions. In contrast, when the probing magnon mode $M_{2}$ is positioned with a π/2 phase shift relative to $M_{1}$, coherent coupling occurs, as shown in Fig. 3C. In this case, the system asymmetry becomes clearly visible in the reflection spectra (Fig. 3, E and G), as both eigenstates contribute to the observed signal. By adjusting the relative positions of the three YIG spheres, we realize an inhomogeneous magnon mode array that can be interpreted as a probing magnon mode $M_{2}$ coupled to a cavity mode supported by the magnonic mirrors, as schematically illustrated in Fig. 4A. The cavity mode corresponds to the dark mode formed by the dissipatively coupled magnon modes $M_{1}$ and $M_{3}$ and is decoupled from the waveguide except through its intrinsic damping rate β. The effective coupling strength between $M_{2}$ and the magnonic cavity mode (dark mode) is

$$J_{D}=\frac{2\sqrt{\kappa_{1}\kappa_{2}\kappa_{3}}}{\sqrt{\kappa_{1}+\kappa_{3}}}$$ (4)

Although the asymmetry between $M_{1}$ and $M_{3}$ induces an additional coupling channel between $M_{2}$ and the bright mode, the bright mode has a much larger linewidth due to its stronger radiative damping. As a result, its quality factor is always lower than that of the dark mode, so its influence can be reasonably ignored in spectroscopic measurements.

### Unidirectional EP of RL states

Following the framework of cavity magnonics (*72*), we can classify the coupling regimes of the MMA system based on the ratio of coupling strength to dissipation, as plotted in Fig. 4B. Since $J_{D}$ depends on the radiative decay rates of $M_{1}$ and $M_{3}$, we can continuously tune the system along the white trajectory in Fig. 4B by adjusting $\kappa_{3}$. With this tunability, we next demonstrate the experimental realization of the unidirectional RL EP.

To clearly observe the unidirectional RL EP and the coupling behaviors between the magnonic mirror cavity and the probing magnon mode, we fix the global magnetic field and bring the $M_{1}$ and $M_{2}$ modes into resonance via a local field $H_{1}$. We then gradually rotate the YIG sphere 3 to effectively vary the frequency detuning $\Delta_{\text{cm}}$ between the magnonic mirror cavity mode and the probing magnon mode. The RL EP condition is verified by extracting the real and imaginary parts of the RL-state eigenvalues $\omega_{\text{RL}}^{l,\pm }$, obtained by directly fitting the reflection spectra ${|S_{11}|}^{2}$ when the magnonic mirror cavity mode and the probing magnon mode are tuned into resonance. When the real part $\text{Re}\left(\omega_{\text{RL}}^{l,\pm }\right)$ and imaginary part $\text{Im}\left(\omega_{\text{RL}}^{l,\pm }\right)$ coalesce simultaneously, the system reaches an EP. By tuning $\kappa_{3}$, we fit a series of reflection spectra to obtain the continuous variation in the eigenvalues, as shown in Fig. 4 (C and D), where the emergence of the RL EP is clearly visible at $\kappa_{3}/2\pi =0.19$ MHz. The RL EP condition is located in the Purcell coupling regime, as marked by a star point in Fig. 4B. It should be noted that ${|S_{22}|}^{2}$ remains a broad reflection peak throughout the variation of $\kappa_{3}$. As a result, fitting the ${|S_{22}|}^{2}$ spectrum to extract the real and imaginary parts of $\omega_{\text{RL}}^{r,\pm }$ is not feasible, since the resonance features are not sufficiently resolved in the measured spectra (see the Supplementary Material for details) (*16*). These results agree well with the theoretical predictions demonstrated in Fig. 2D. At the RL EP, the coupling strength matches the linewidth of $M_{2}$, preventing spectral splitting of the hybridized modes. This is identified in the reflection spectra measured through waveguide port one (Fig. 4E), where a broadened spectrum is observed at resonance.

Figure 4G presents the reflection spectra measured at resonance for signals incident from both ports, revealing pronounced directional behavior. A clear unidirectional response is observed, with the reflection from port one exhibiting an almost RL dip (${|S_{11}|}^{2}$), while the reflection from port two remains near unity. It is worth noting that this result shows a flattened quartic line shape in the linear scale plot in Fig. 4H, which is a distinct signature of the EP. The mechanisms can be understood as follows. When excitation is launched from the high-reflectivity magnonic mirror ($M_{1}$, i.e., port two), the signal is predominantly reflected and fails to access the MMA. Conversely, when incident from the low-reflectivity magnonic mirror ($M_{3}$, i.e., port one), the microwave signal couples into the MMA and interacts with the probing magnon mode ($M_{2}$), producing a distinct RL valley.

The RL EP behavior is further validated by enhancing the coupling strength to shift the system into the strong coupling regime. As shown in Fig. 4F, when the magnonic mirror cavity mode resonates with the probing magnon mode, the hybridized polariton modes exhibit a clear spectral splitting. The system’s unidirectionality is preserved, and two well-separated Lorentzian dips are observed in the port-one reflection spectrum at resonance (Fig. 4I). The bandwidth of one Lorentizian dip is substantially narrower than that of the quartic line shape at the RL EP (Fig. 4J), leading to the reduction of the unidirectional RL bandwidth. The asymmetry in the reflection spectra on a linear scale in Fig. 4 (H and J) arises from the Fano interference introduced by the GSE. This effect does not compromise the appearance of the quartic dip. While such mode splitting has been observed previously (*54*), the non-Hermitian asymmetry engineered in our system enables its observation from a single direction. This unidirectional accessibility opens up possibilities for developing direction-selective photonic devices in open waveguide systems.

## DISCUSSION

In conclusion, we have proposed and experimentally demonstrated a unidirectional RL EP in an MMA system, enabled by a collective dark state. A GSE with three spatially separated coupling points is used as a tunable magnonic mirror, providing an effective method to control the system’s asymmetry. This configuration results in a substantial enhancement of the magnon’s radiative damping and enables the emergence of unidirectional RL behavior. By finely tuning the detuning and coupling strengths of the magnon modes, we observe the coalescence of RL eigenfrequencies in one direction, manifested as a flattened quartic reflection dip, which is an unambiguous signature of the RL EP. This distinctive line shape is beneficial for applications such as stealth and energy harvesting technologies (*22*).

The evolution of unidirectional RL states also indicates coherent interactions among the hybridized modes in the MMA system. The RL dip in the ${|S_{11}|}^{2}$ reflection spectrum bifurcates beyond the EP as the coupling strength $J_{D}$ increases, signifying the transition of the equivalent magnonic mirror cavity system into the strong-coupling regime. The unidirectional RL state can thus provide an opportunity to directly observe MMA polariton behaviors via waveguide transmission in a single direction. This directionality persists until the system regains inversion symmetry (see the Supplementary Materials for details).

This work not only enriches the understanding of RL EPs in non-Hermitian asymmetric systems but also establishes a GSE-based reconfigurable and scalable platform for manipulating directional wave control, thereby broadening the scope of GSE applications. The GSE can be readily implemented on lithographic platforms using broadband, high transmission microstrip structures. By adopting analogous meandering waveguide strategies (*84*) and appropriate emitters [e.g., excitation modes in antiferromagnets (*85*, *86*) and quantum dots (*87*, *88*)], the approach can naturally be extended to the terahertz and optical frequency domains. The demonstrated architecture is also compatible with integrated microwave photonics and can be scaled to complex magnonic lattices or hybrid quantum systems.

## MATERIALS AND METHODS

### Device design

The meandering waveguide shown in Fig. 1D is fabricated on a 0.813-mm-thick RO4003C substrate with a 1-oz copper conductor. To achieve the GSE with three coupling points, the microstrip with a width of 1.82 mm is connected with miter corners. The 1-mm-diameter YIG spheres that we use are commercially available.

### Measurement and parameter tuning

The reflection are measured using a vector network analyzer (KEYSIGHT PNA-L Network Analyzer N5232B) with an input power of −10 dBm. The resonance frequency of the $M_{3}$ mode can be independently tuned by changing the anisotropic field, which depends on the angle $\theta_{H}$ between the crystal axis and the applied field (*35*). We implement this by mounting $M_{3}$ on a cantilever driven by a rotary motor, allowing angular adjustment via spatial translation. As shown in Fig. 1F, this enables continuous tuning of $\omega_{3}/2\pi$ approximately more than a 300-MHz range. The regulation of $\kappa_{3}$ is achieved by translating the green sphere along the *z* axis (Fig. 1E), with extracted results displayed in Fig. 1G. Since $M_{1,2,3}$ are identical YIG spheres, their radiative damping rates into the waveguide should be equal under identical coupling conditions. In our setup, $M_{3}$ is intentionally placed farther from the microstrip, so its radiative damping rate is smaller than $M_{2}$. The $M_{1}$ is designed as a GSE to have the greater radiation due to the constructive interference between the coupling points. Their difference is clearly evident in the corresponding transmission spectra.

## Supplementary Materials

### This PDF file includes:

Supplementary Text Figs. S1 to S8 References
