# Supplementary material for: Unidirectional exceptional point of reflectionless states in a magnonic mirror array
Source: Sci Adv. 2026 Mar 4;12(10):eaea6000. doi: 10.1126/sciadv.aea6000 (PMC12959398; doi:10.1126/sciadv.aea6000)
Supplement: Supplementary file 1 — Supplementary Text Figs. S1 to S8 References [file sciadv.aea6000_sm.pdf]

Science Advances
AAAS

Supplementary Materials for

Unidirectional exceptional point of reflectionless states in a magnonic mirror array

Zi-Qi Wang *et al.*

Corresponding author: Yi-Pu Wang, yipuwang@zju.edu.cn; J. Q. You, jqyou@zju.edu.cn

Sci. Adv. **12**, eaea6000 (2026)
DOI: 10.1126/sciadv.aea6000

This PDF file includes:

Supplementary Text
Figs. S1 to S8
References

Supplementary Text

S1. Exceptional Points of the Scattering Matrix and Reflectionless States

A scattering exceptional point (EP) arises at specific locations in the parameter space where the non-Hermitian scattering matrix S becomes defective, i.e., its eigenvalues and eigenvectors coalesce. For a reciprocal two-port system, the scattering matrix can be written as

$$S = \begin{pmatrix} T_l & R_l \\ R_r & T_r \end{pmatrix}, \tag{S1}$$

where T and R denote the transmission and reflection coefficients, respectively, with subscripts indicating the direction of signal incidence. For example, R_l (T_l) represents the reflection (transmission) coefficient for a signal incident from the left port.

In a reciprocal system ($T_l = T_r = t$), the eigenvalues of the S-matrix are given by

$$\omega_S^{\pm} = t \pm \sqrt{R_l R_r}. \tag{S2}$$

An EP is reached when the square-root term vanishes, i.e., when the product $R_l R_r = 0$. This condition typically yields multiple frequencies $\omega_{\mathrm{RL}}^{(1)}, \omega_{\mathrm{RL}}^{(2)}, \ldots$ corresponding to reflectionless (RL) states. A further degeneracy of these RL states defines a reflectionless exceptional point (RL EP). Previous studies have primarily focused on RL EPs emerging in systems with inversion symmetry, where both reflection coefficients vanish simultaneously ($R_l = R_r = 0$), leading to bidirectional RL EPs. However, RL EPs can also occur in systems without inversion symmetry. For example, when $R_l = 0$ and $R_r \neq 0$, the system allows for the realization of *unidirectional* RL EPs.

S2. Effective Hamiltonian of a GSE-Waveguide System

In this section, we consider a more general case in which multiple giant spin ensembles (GSEs) interact with a meandering microwave microstrip waveguide through arbitrary coupling points, as illustrated in Supplementary Fig. S1. The magnon mode we only concern in this work is the Kittel mode. The Kittel mode in each YIG sphere interacts with both right- and left-propagating photon modes in the waveguide. The total Hamiltonian of the GSE-waveguide system is given by $\hat{H} = \hat{H}_{\mathrm{wg}} + \hat{H}_{\mathrm{m}} + \hat{H}_{\mathrm{int}}$, where $\hat{H}_{\mathrm{wg}}$, $\hat{H}_{\mathrm{m}}$, and $\hat{H}_{\mathrm{int}}$ describe the waveguide, the magnon modes, and their mutual interaction, respectively. In the following analysis, we set $\hbar = 1$ for simplicity. We consider

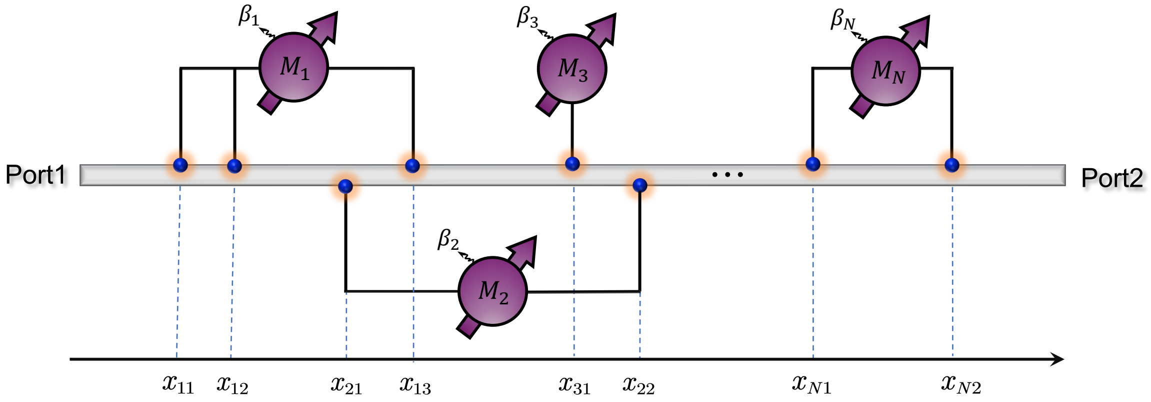

Figure S1: **Schematic of the GSE–waveguide system.** Multiple YIG spheres are coupled to the waveguide at arbitrary positions. The waveguide has two ports labeled Port 1 and Port 2.

N GSEs, where the ith GSE couples to the waveguide through P_i spatially separated points. Under the rotating-wave approximation, the real-space Hamiltonian of the system reads (*30, 89*)

$$\hat{H}_{\mathrm{m}} = \sum_{i=1}^{N} (\omega_i - i\beta_i)\hat{m}_i^{\dagger}\hat{m}_i, \tag{S3}$$

$$\hat{H}_{\mathrm{wg}} = -iv_{\mathrm{g}} \int dx \left[\hat{c}_r^{\dagger}(x)\frac{\partial}{\partial x}\hat{c}_r(x) - \hat{c}_l^{\dagger}(x)\frac{\partial}{\partial x}\hat{c}_l(x)\right], \tag{S4}$$

$$\hat{H}_{\mathrm{int}} = \int dx \sum_{i=1}^{N}\sum_{p=1}^{P_i} g_{ip}\delta(x - x_{ip}) \left[\hat{c}_r^{\dagger}(x)\hat{m}_i + \hat{c}_l^{\dagger}(x)\hat{m}_i + \mathrm{H.c.}\right]. \tag{S5}$$

Here ω_i and β_i denote the frequency and intrinsic loss rate of the Kittel mode in the ith YIG sphere, which follows $\omega_i = \gamma(H_{e,i} + H_A)$, where $\gamma/2\pi = 28$ GHz/T is the gyromagnetic ratio and H_A is the anisotropy field. The operator $\hat{m}_i^{\dagger}$ ($\hat{m}_i$) creates (annihilates) a magnon excitation in the ith GSE. The operators $\hat{c}_r^{\dagger}(x)$ and $\hat{c}_l^{\dagger}(x)$ [$\hat{c}_r(x)$ and $\hat{c}_l(x)$] create (annihilate) right- and left-propagating photons at position x, respectively. The coordinate x_{ip} denotes the position of the pth coupling point of the ith GSE and v_{g} is the photon group velocity in the waveguide. The parameter g_{ip} denotes the coupling strength between the waveguide and the ith GSE at the pth coupling point.

In the single-excitation subspace, the eigenstate of the system can be written as

$$|\Psi\rangle = \int dx \left[\Phi_r(x)\hat{c}_r^{\dagger}(x) + \Phi_l(x)\hat{c}_l^{\dagger}(x)\right]|\emptyset\rangle + \sum_{i=1}^{N} f_i\hat{m}_i^{\dagger}|\emptyset\rangle, \tag{S6}$$

where $|\emptyset\rangle$ is the vacuum state (all modes unexcited), $\Phi_r(x)$ $[\Phi_l(x)]$ is the wavefunction for a rightward (leftward) propagating photon and f_i is the excitation amplitude of the magnon mode. Assume a single input photon with frequency $\omega = v_g k$ is incident from the left (Port 1), where k is the momentum of the photon. The photon wavefunction ansatz reads

$$\Phi_r(x) = e^{ikx}\left[\theta(x_1 - x) + \sum_{s=1}^{N_c-1} t_s\theta(x - x_s)\theta(x_{s+1} - x) + t\theta(x - x_{N_c})\right], \tag{S7a}$$

$$\Phi_l(x) = e^{-ikx}\left[r\theta(x_1 - x) + \sum_{s=2}^{N_c} r_s\theta(x - x_{s-1})\theta(x_s - x)\right], \tag{S7b}$$

where x_s are the ordered positions of all coupling points, and $N_c = \sum_{i=1}^{N} P_i$. Here, T_s (R_s) denotes the local transmission (reflection) amplitude in each segment, and $\theta(x)$ is the Heaviside function. By substituting Eq. (S6) into the Schrödinger equation $\hat{H}|\Psi\rangle = \omega|\Psi\rangle$, and following the method in Ref. (*44*), we obtain the transmission and reflection coefficients

$$T = 1 - i\mathbf{V}^\dagger(\omega\mathbf{I} - H_{\text{eff}})^{-1}\mathbf{V}, \tag{S8a}$$

$$R = -i\mathbf{V}^\top(\omega\mathbf{I} - H_{\text{eff}})^{-1}\mathbf{V}. \tag{S8b}$$

Here, $\mathbf{f} = (f_1, f_2, \ldots, f_N)^\top$, and $\mathbf{V} = (\mathcal{V}_1, \mathcal{V}_2, \ldots, \mathcal{V}_N)^\top$, where

$$\mathcal{V}_i = \sum_{p=1}^{P_i} \sqrt{\kappa_{ip}} e^{i\varphi_{ip}}, \tag{S9}$$

with $\kappa_{ip} = g_{ip}^2/v_g$ being the decay rate into the waveguide, and $\varphi_{ip} = \omega_r x_{ip}/v_g$ is the phase at reference frequency ω_r (valid in the Markovian regime).

The effective non-Hermitian Hamiltonian is given by

$$\mathcal{H}_{ij} = (\omega_i - i\beta_i)\delta_{ij} - i\sum_{p=1}^{P_i}\sum_{p'=1}^{P_j} \sqrt{\kappa_{ip}\kappa_{jp'}} e^{i|\varphi_{ip} - \varphi_{jp'}|}. \tag{S10}$$

Alternatively, it can be decomposed as

$$\mathcal{H}_{ij} = \begin{cases} \omega_i + \Delta_{\text{L},i} - i(\beta_i + \kappa_{\text{eff},i}) & \text{for } i = j, \\ J_{ij} - \frac{i}{2}\Gamma_{ij} & \text{for } i \neq j, \end{cases} \tag{S11}$$

where

$$\Delta_{\mathrm{L},i} = \sum_{p,p'} \sqrt{\kappa_{ip}\kappa_{ip'}} \sin|\varphi_{ip} - \varphi_{ip'}|, \tag{S12a}$$

$$\kappa_{\mathrm{eff},i} = \sum_{p,p'} \sqrt{\kappa_{ip}\kappa_{ip'}} \cos(\varphi_{ip} - \varphi_{ip'}). \tag{S12b}$$

The coherent and dissipative couplings between distinct magnon modes are

$$J_{ij} = \sum_{p}^{P_i} \sum_{p'}^{P_j} \sqrt{\kappa_{ip}\kappa_{jp'}} \sin|\varphi_{ip} - \varphi_{jp'}|, \tag{S13a}$$

$$\Gamma_{ij} = 2\sum_{p}^{P_i} \sum_{p'}^{P_j} \sqrt{\kappa_{ip}\kappa_{jp'}} \cos(\varphi_{ip} - \varphi_{jp'}). \tag{S13b}$$

These results are general and apply not only to GSEs each with multiple coupling points but also to conventional spin ensembles where $P_i = 1$. All scattering spectra illustrated throughout this work are calculated using Eqs. (S8a) and (S8b).

S3. Effectively enhanced radiative decay rate of a GSE

In this section, we consider the case in which only a single GSE or spin ensemble is coupled to the waveguide. This corresponds to $N = 1$, and we denote the magnon frequency as ω_{m}. Equations (S9) and (S10) are simplified to

$$V = \sum_{p=1}^{P} \sqrt{\kappa_p} e^{i\varphi_p}, \tag{S14}$$

$$H = \omega_{\mathrm{m}} + \Delta_{\mathrm{L}} - i(\beta + \kappa_{\mathrm{eff}}), \tag{S15}$$

where P is the number of coupling points of the single GSE. If we assume identical radiative decay rates $\kappa_p = \kappa_0$ for all coupling points and a constant phase difference φ_0 between adjacent ones, i.e., $\varphi_p = (p-1)\varphi_0$, then we have

$$\Delta_{\mathrm{L}} = \kappa_0 \sum_{p=1}^{P} \sum_{p'=1}^{P} \sin|(p-p')\varphi_0| = \kappa_0 \frac{P\sin\varphi_0 - \sin(P\varphi_0)}{1-\cos\varphi_0}, \tag{S16}$$

$$\kappa_{\mathrm{eff}} = \kappa_0 \sum_{p=1}^{P} \sum_{p'=1}^{P} \cos[(p-p')\varphi_0] = \kappa_0 \frac{1-\cos(P\varphi_0)}{1-\cos\varphi_0}. \tag{S17}$$

According to Eq. (S17), in the limit $\varphi_0 \to 2m\pi$ with $m \in \mathbb{Z}$, applying L'Hôpital's rule yields $\kappa_{\text{eff}} = P^2\kappa_0$. Thus, adding more coupling points provides an effective route to boost the spin ensemble's radiation. By substituting these expressions into Eqs. (S8a) and (S8b), the transmission and reflection coefficients take the following forms:

$$T = 1 - i\frac{\kappa_0 \sum_{p=1}^{P} \sum_{p'=1}^{P} e^{i(p'-p)\varphi_0}}{\omega - \omega_{\text{m}} - \Delta_{\text{L}} + i(\beta + \kappa_{\text{eff}})} = \frac{\omega - \omega_{\text{m}} - \Delta_{\text{L}} + i\beta}{\omega - \omega_m - \Delta_{\text{L}} + i(\beta + \kappa_{\text{eff}})}, \tag{S18a}$$

$$R = -i\frac{\kappa_0 \sum_{p=1}^{P} \sum_{p'=1}^{P} e^{i(p'+p)\varphi_0}}{\omega - \omega_{\text{m}} - \Delta_{\text{L}} + i(\beta + \kappa_{\text{eff}})} = \frac{\kappa_{\text{eff}} e^{-i2\alpha}}{\omega - \omega_{\text{m}} - \Delta_{\text{L}} + i(\beta + \kappa_{\text{eff}})}, \tag{S18b}$$

where the phase factor α satisfies

$$\tan(2\alpha) = \frac{\sum_{p,p'=1}^{P} \sin[(p + p')\varphi_0]}{\sum_{p,p'=1}^{P} \cos[(p + p')\varphi_0]}. \tag{S19}$$

From Eqs. (S18a) and (S18b), it is clear that regardless of the number of coupling points, the transmission and reflection spectra of a single GSE always exhibit a Lorentzian lineshape centered at $\omega = \omega_{\text{m}} + \Delta_{\text{L}}$, with linewidth κ_{eff}. Therefore, by tailoring the number of coupling points P and the phase offset φ_0, the effective radiative decay rate κ_{eff} can be precisely engineered and significantly enhanced relative to κ_0, as illustrated in Supplementary Fig. S2.

This result also indicates that a spatially distributed GSE with multiple coupling points can be reduced to a single spin ensemble. Since the main text focuses on spatially separated GSEs, in the following discussion, we treat each GSE as an spin ensemble with $P_i = 1$ for simplicity.

S4. Reciprocal Transmission and Asymmetric Reflection

From Eqs. (S8a) and (S8b), it is evident that the effective Hamiltonian H_{eff} governs the scattering behavior of the GSE-waveguide system. Being non-Hermitian, H_{eff} can be diagonalized as $H_{\text{eff}} = \sum_n E_n |\Psi_n^R\rangle\langle\Psi_n^L|$, where $\langle\Psi_n^L|\Psi_{n'}^R\rangle = \delta_{nn'}$ defines the normalized biorthogonal basis. Consequently, the transmission and reflection amplitudes can be expressed in terms of the collective modes of the GSEs as

$$T(\omega) = 1 - i\sum_{n=1}^{N} \frac{\mathbf{V}^\dagger|\Psi_n^R\rangle\langle\Psi_n^L|\mathbf{V}}{\omega - \text{Re}(E_n) - i\text{Im}(E_n)}, \tag{S20a}$$

$$R(\omega) = -i\sum_{n=1}^{N} \frac{\mathbf{V}^\top|\Psi_n^R\rangle\langle\Psi_n^L|\mathbf{V}}{\omega - \text{Re}(E_n) - i\text{Im}(E_n)}. \tag{S20b}$$

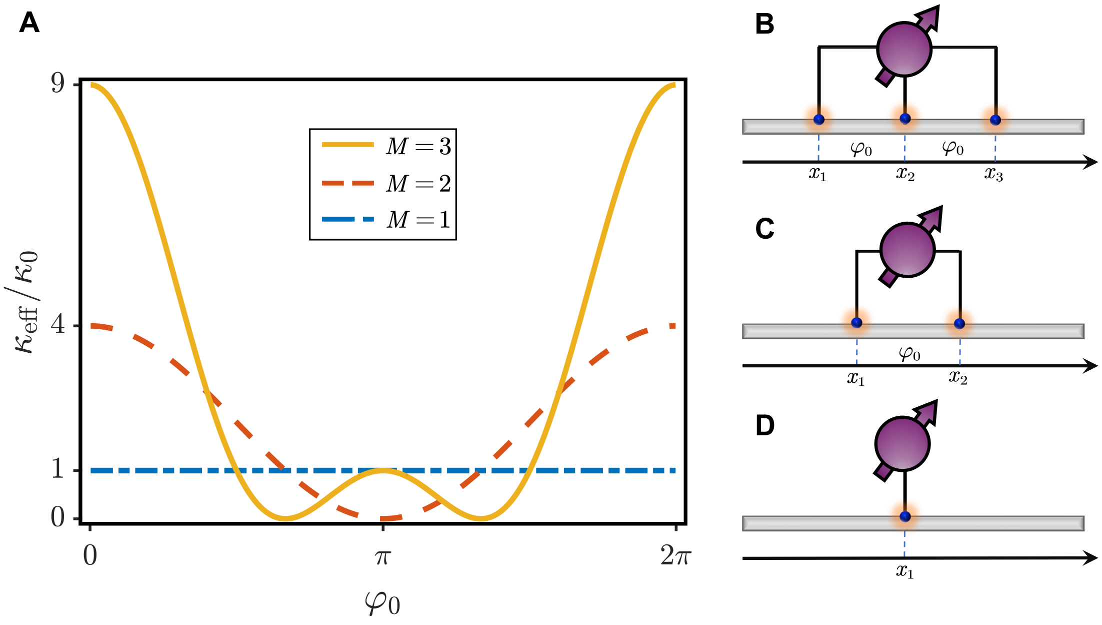

Figure S2: **Effective decay rate of a single GSE.** (**A**) Variation of the effective decay rate with phase difference between coupling points. Schematics for cases with (**B**) three, (**C**) two, and (**D**) one coupling points are shown.

These expressions show that the scattering process can be understood as an interference of multiple Lorentzian modes, each corresponding to a collective eigenmode of H_{eff} with center frequency $\text{Re}(E_n)$ and linewidth $\text{Im}(E_n)$. Each mode contributes a distinct scattering channel. The biorthogonal eigenstates of the non-Hermitian Hamiltonian are defined as

$$H_{\text{eff}}|\Psi_n^R\rangle = E_n|\Psi_n^R\rangle, \tag{S21a}$$

$$H_{\text{eff}}^\dagger|\Psi_n^L\rangle = E_n^*|\Psi_n^L\rangle. \tag{S21b}$$

Due to the symmetry $H_{\text{eff}} = H_{\text{eff}}^\top$, it follows that $H_{\text{eff}}^\dagger = (H_{\text{eff}}^\top)^* = H_{\text{eff}}^*$, and Eq. (S21b) becomes

$$H_{\text{eff}}^*|\Psi_n^L\rangle = E_n^*|\Psi_n^L\rangle. \tag{S22}$$

By comparing with the complex conjugate of Eq. (S21a), we obtain

$$|\Psi_n^L\rangle = (|\Psi_n^R\rangle)^*, \quad \langle\Psi_n^L| = (\langle\Psi_n^R|)^*. \tag{S23}$$

In Eqs. (S20a) and (S20b), the numerators are defined as coupling efficiency terms, which quantify the excitation efficiency of the collective eigenmodes of H_{eff} by the incident photon mode, i.e., the overlap between the input field and each eigenmode of the system (*90*). Using Eq. (S23), the interaction spectrum can be expressed solely in terms of the right eigenstates

$$\tilde{\eta}_n = \mathbf{V}^\dagger |\Psi_n^R\rangle \cdot \mathbf{V}^\top |\Psi_n^R\rangle, \tag{S24a}$$

$$\eta_n = \mathbf{V}^\top |\Psi_n^R\rangle \cdot \mathbf{V}^\top |\Psi_n^R\rangle. \tag{S24b}$$

To investigate the scattering properties, we consider photons incident from the right (Port 2), which corresponds to reversing the propagation direction ($k \to -k$), resulting in $\mathbf{V} \to \mathbf{V}^*$. The port-resolved scattering matrix elements are defined as

$$T^l \equiv S_{21} = 1 - i\sum_{n=1}^{N} \frac{\mathbf{V}^\dagger |\Psi_n^R\rangle \cdot \mathbf{V}^\top |\Psi_n^R\rangle}{\omega - E_n} \equiv 1 - i\sum_{n=1}^{N} \frac{\tilde{\eta}_n^{(l)}}{\omega - E_n}, \tag{S25a}$$

$$T^r \equiv S_{12} = 1 - i\sum_{n=1}^{N} \frac{\mathbf{V}^\top |\Psi_n^R\rangle \cdot \mathbf{V}^\dagger |\Psi_n^R\rangle}{\omega - E_n} \equiv 1 - i\sum_{n=1}^{N} \frac{\tilde{\eta}_n^{(r)}}{\omega - E_n}, \tag{S25b}$$

$$R^l \equiv S_{11} = -i\sum_{n=1}^{N} \frac{\mathbf{V}^\top |\Psi_n^R\rangle \cdot \mathbf{V}^\top |\Psi_n^R\rangle}{\omega - E_n} \equiv -i\sum_{n=1}^{N} \frac{\eta_n^{(l)}}{\omega - E_n}, \tag{S25c}$$

$$R^r \equiv S_{22} = -i\sum_{n=1}^{N} \frac{\mathbf{V}^\dagger |\Psi_n^R\rangle \cdot \mathbf{V}^\dagger |\Psi_n^R\rangle}{\omega - E_n} \equiv -i\sum_{n=1}^{N} \frac{\eta_n^{(r)}}{\omega - E_n}. \tag{S25d}$$

The superscripts indicate photons incident from the left and right, respectively. To avoid ambiguity, the transmission and reflection coefficients are redefined using uppercase letters. From these expressions, it is evident that $\tilde{\eta}_n^{(l)} \neq \tilde{\eta}_n^{(r)}$ or $\eta_n^{(l)} \neq \eta_n^{(r)}$ are necessary conditions for realizing nonreciprocal transmission or symmetric reflection. In the absence of time-reversal symmetry breaking, the coupling amplitudes from the left and right satisfy $\tilde{\eta}_n^{(l)} = \tilde{\eta}_n^{(r)}$, which leads to reciprocal transmission, i.e., $S_{21} = S_{12}$. However, for reflection, the interaction terms generally satisfy $\eta_n^{(l)} \neq \eta_n^{(r)}$, except in special cases where $\mathbf{V}^\dagger = \mathbf{V}^\top$ or the system exhibits spatial inversion symmetry. In these cases, symmetric reflection is recovered, while in all other situations, reflection asymmetry arises with $S_{11} \neq S_{22}$. In the following, we focus on analyzing the asymmetry in the reflection spectrum.

S5. Reflectionless Hamiltonian of a two-port waveguide magnonic system

To reveal the condition for the coalescence of the RL states, the reflectionless Hamiltonian H_{RL} should be dervied analytically. We consider a $N \times N$ effective Hamiltonian H_{eff} with coupling vector $\mathbf{V} = (v_1, v_2, \ldots, v_N) \in \mathbb{C}^{1\times N}$. The reflection coefficient is given by

$$R(\omega) = -i\,\mathbf{V}\,(\,\omega\mathbf{I}_N - H_{\mathrm{eff}}\,)^{-1}\mathbf{V}^{\top}. \tag{S26}$$

Obviously, the above expression does not clearly indicate the condition under which the reflection coefficient vanishes. To resolve this issue, we transforms the reflection coefficient into a determinant ratio in which the zeros are dictated solely by the eigenvalues of the reflectionless Hamiltonian H_{RL}. This formula is widely used in other studies on EP arising in scattering (*17, 18, 20*), which provides a transparent way to uncover the physical mechanism and the conditions for degeneracy.

Because the expression in Eq. (S26) differs from that of a one-port system (*17*), we cannot use a similar mathematical approach to obtain H_{RL} in a simple way. Here, we provide a method for solving the H_{RL} in a two-port system by performing an orthogonal transformation that aligns the vector $\mathbf{V}$ onto the last coordinate axis. Explicitly, there exists $\mathbf{S} \in O(N, \mathbb{C})$ such that

$$\mathbf{S}\,\mathbf{V}^{\top} = \sqrt{\mathbf{V}\mathbf{V}^{\top}}\,\mathbf{e}_N, \tag{S27}$$

where $\mathbf{e}_N$ is the Nth canonical basis vector. An explicit construction is obtained by defining

$$\mathbf{w} = \mathbf{V}^{\top} - \sqrt{\mathbf{V}\mathbf{V}^{\top}}\,\mathbf{e}_N, \tag{S28}$$

and taking

$$\mathbf{S} = \mathbf{I}_N - \frac{2\,\mathbf{w}\mathbf{w}^{\top}}{\mathbf{w}^{\top}\mathbf{w}}. \tag{S29}$$

This $\mathbf{S}$ satisfies $\mathbf{S}^{\top}\mathbf{S} = \mathbf{I}_N$ and rotates $\mathbf{V}^{\top}$ exactly onto the last axis as required by Eq. (S27). Writing $\mathbf{M}(\omega) = \omega\mathbf{I}_N - H_{\mathrm{eff}}$ and $\mathbf{M}'(\omega) = \mathbf{S}\,\mathbf{M}(\omega)\,\mathbf{S}^{\top}$, Eq. (S26) becomes

$$R(\omega) = -i\,(\mathbf{V}\mathbf{V}^{\top})\,[\mathbf{M}'(\omega)^{-1}]_{NN}. \tag{S30}$$

From the adjugate formula,

$$[\mathbf{M}'(\omega)^{-1}]_{NN} = \frac{\det(\mathbf{M}'_{(N-1)\times(N-1)}(\omega))}{\det\mathbf{M}(\omega)}. \tag{S31}$$

Thus the reflection coefficient reduces to

$$R(\omega) = -i\,(\mathbf{V}\mathbf{V}^{\top})\,\frac{\det(\mathbf{M}'_{(N-1)\times(N-1)}(\omega))}{\det(\,\omega\mathbf{I}_N - \mathbf{H}_{\text{eff}}\,)}. \tag{S32}$$

If $\mathbf{Q} \in \mathbb{C}^{N\times(N-1)}$ is an orthonormal basis with $\mathbf{VQ} = 0$, then

$$\mathbf{M}'_{(N-1)\times(N-1)}(\omega) = \mathbf{Q}^{\top}\mathbf{M}(\omega)\mathbf{Q} = \omega\mathbf{I}_{N-1} - \mathbf{Q}^{\top}\mathbf{H}_{\text{eff}}\mathbf{Q}. \tag{S33}$$

Here $\mathbf{Q}$ can be chosen explicitly as the first $N-1$ columns of $\mathbf{S}^{\top}$, where $\mathbf{S}$ is the complex orthogonal matrix defined in Eq. (S27). This motivates the definition of the reflectionless Hamiltonian as

$$\mathbf{H}_{\text{RL}} = \mathbf{Q}^{\top}\mathbf{H}_{\text{eff}}\mathbf{Q}. \tag{S34}$$

Accordingly, the reflection coefficient can be expressed as

$$R(\omega) = -i\,(\mathbf{V}\mathbf{V}^{\top})\,\frac{\det(\omega\mathbf{I}_{N-1} - \mathbf{H}_{\text{RL}})}{\det(\omega\mathbf{I}_N - \mathbf{H}_{\text{eff}})}. \tag{S35}$$

Hence the RL states are determined exactly by the eigenvalues of $\mathbf{H}_{\text{RL}}$. It should be noted that this compact expression is valid only when $\mathbf{V}\mathbf{V}^{\top} \neq 0$. Therefore, $\mathbf{V}\mathbf{V}^{\top} = 0$ does *not* imply $R(\omega) \equiv 0$. If $\mathbf{V}\mathbf{V}^{\top} = 0$, Eq. (S35) becomes ill-defined, but the original definition in Eq. (S26) remains valid and generally yields nonzero values.

S6. Effective and Reflectionless Hamiltonian of an Anti-Bragg magnon array

To present the RL Hamiltonian of our system, we first derive the corresponding effective Hamiltonian. Base on the formula in Section S2, the effective Hamiltonian describing three magnon modes coupled to a waveguide can be written as

$$H_{\text{eff}} = \begin{pmatrix} \omega_{\text{m}} - i(\kappa_1 + \beta) & -i\sqrt{\kappa_1\kappa_2}\,e^{i\frac{2\pi d_{12}}{\lambda_{\text{m}}}} & -i\sqrt{\kappa_1\kappa_3}\,e^{i\frac{2\pi d_{13}}{\lambda_{\text{m}}}} \\ -i\sqrt{\kappa_1\kappa_2}\,e^{i\frac{2\pi d_{12}}{\lambda_{\text{m}}}} & \omega_{\text{m}} - i(\kappa_2 + \beta) & -i\sqrt{\kappa_2\kappa_3}\,e^{i\frac{2\pi d_{23}}{\lambda_{\text{m}}}} \\ -i\sqrt{\kappa_1\kappa_3}\,e^{i\frac{2\pi d_{13}}{\lambda_{\text{m}}}} & -i\sqrt{\kappa_2\kappa_3}\,e^{i\frac{2\pi d_{23}}{\lambda_{\text{m}}}} & \omega_{\text{m}} - i(\kappa_3 + \beta) \end{pmatrix}. \tag{S36}$$

Here we consider all magnon modes to be resonant at the same frequency ω_{m}, with an identical intrinsic damping rate β. The spatial separation between the ith and jth magnon modes is denoted as $d_{ij} = |x_i - x_j|$, κ_i ($i = 1, 2, 3$) represents the radiative decay rate of each magnon mode, and λ_{m} is the wavelength of the traveling photon mode at the magnon resonance frequency. In the

following, we assume that the magnon modes are equally spaced, i.e., $d_{ij} = d|i-j|$, where d is the distance between adjacent magnon modes. The phase term $e^{i2\pi d|i-j|/\lambda_{\mathrm{m}}}$ directly influences the coupling among the magnon modes and the reflection spectra of the system. As two specific spatial configurations, the Bragg and anti-Bragg arrays yield totally different reflection behaviors. The Bragg (anti-Bragg) condition is defined by the spatial separation d between spin ensembles as

$$d = n\lambda_m/4, \tag{S37}$$

where n is an even (odd) integer. Substituting Eq. (S37) into Eq. (S9), the coupling vector $\mathbf{V}$ can be expressed as

$$\mathbf{V} = (\sqrt{\kappa_1}, \sqrt{\kappa_2}e^{i\frac{\pi n}{2}}, \sqrt{\kappa_3}e^{i\pi n}, \cdots, \sqrt{\kappa_N}e^{i\frac{\pi n(N-1)}{2}}). \tag{S38}$$

When the magnon array satisfy the Bragg condition (n is even), the relation

$$\mathbf{V}^{\top}_{\text{Bragg}} = \mathbf{V}^{\dagger}_{\text{Bragg}} \tag{S39}$$

holds, implying that left- and right-incident coupling vector are identical. Consequently, the reflection spectra are always symmetric under Bragg condition. This is because only the superradiance state can be detected in this system (*31*). In our experiment, this property enables direct determination of relative phase between spin ensembles. For example, a π phase difference between the first and third spin ensembles is verified by the symmetric reflection spectra shown in Figs. 3D and 3F of the main text.

In contrast, under the anti-Bragg condition (odd n), the relation $\mathbf{V}^{\top} = \mathbf{V}^{\dagger}$ no longer holds, resulting in asymmetric reflection. In our case, a $\pi/2$ phase difference between the first and second spin ensembles is confirmed by the asymmetric spectra shown in Figs. 3E and 3G of the main text. This is the key mechanism enabling the system to support unidirectional RL states. Under the anti-Bragg configuration, Eq. (S36) reduces to

$$H_{\text{eff}}^{\text{anti-Bragg}} = \begin{pmatrix} \omega_{\mathrm{m}} - i(\kappa_1+\beta) & \sqrt{\kappa_1\kappa_2} & i\sqrt{\kappa_1\kappa_3} \\ \sqrt{\kappa_1\kappa_2} & \omega_{\mathrm{m}} - i(\kappa_2+\beta) & \sqrt{\kappa_2\kappa_3} \\ i\sqrt{\kappa_1\kappa_3} & \sqrt{\kappa_2\kappa_3} & \omega_{\mathrm{m}} - i(\kappa_3+\beta) \end{pmatrix}, \tag{S40}$$

where coherent coupling occurs between nearest-neighbor magnon modes, while dissipative coupling arises between next-nearest neighbors. Using the mathematical formula introduced in Sec. S5,

we can obtain the RL Hamiltonian under the anti-Bragg condition. For right-incident input,

$$\mathbf{V}^{(r)} = (\sqrt{\kappa_1},\ i\sqrt{\kappa_2},\ -\sqrt{\kappa_3}), \qquad \mathbf{V}^{(r)}\big(\mathbf{V}^{(r)}\big)^\top = \kappa_1 - \kappa_2 + \kappa_3, \tag{S41}$$

and for left incidence,

$$\mathbf{V}^{(l)} = (\sqrt{\kappa_1},\ -i\sqrt{\kappa_2},\ -\sqrt{\kappa_3}) = \big(\mathbf{V}^{(r)}\big)^*, \qquad \mathbf{V}^{(l)}\big(\mathbf{V}^{(l)}\big)^\top = \kappa_1 - \kappa_2 + \kappa_3. \tag{S42}$$

Following Eqs. (S28)–(S29), we define for each incidence direction

$$\mathbf{w}^{(r)} = \begin{pmatrix} \sqrt{\kappa_1} \\ i\sqrt{\kappa_2} \\ -\sqrt{\kappa_3} - \sqrt{\kappa_1 - \kappa_2 + \kappa_3} \end{pmatrix}, \qquad \mathbf{w}^{(l)} = \begin{pmatrix} \sqrt{\kappa_1} \\ -i\sqrt{\kappa_2} \\ -\sqrt{\kappa_3} - \sqrt{\kappa_1 - \kappa_2 + \kappa_3} \end{pmatrix}, \tag{S43}$$

and construct

$$\mathbf{S}^{r(l)} = \mathbf{I}_3 - \frac{\mathbf{w}^{r(l)}\big(\mathbf{w}^{r(l)}\big)^\top}{\kappa_1 - \kappa_2 + \kappa_3 + \sqrt{\kappa_3}\sqrt{\kappa_1 - \kappa_2 + \kappa_3}}. \tag{S44}$$

This satisfies $\big(\mathbf{S}^{r(l)}\big)^\top \mathbf{S}^{r(l)} = \mathbf{I}_3$ and $\mathbf{S}^{r(l)}\big(\mathbf{V}^{r(l)}\big)^\top = \sqrt{\kappa_1 - \kappa_2 + \kappa_3}\,\mathbf{e}_3$. Taking the first two columns of $\big(\mathbf{S}^{r(l)}\big)^\top$ as $\mathbf{Q}^{r(l)}$, the reduced RL Hamiltonian is obtained as

$$H_{\mathrm{RL}}^{r(l)} = \big(\mathbf{Q}^{r(l)}\big)^\top H_{\mathrm{eff}}\,\mathbf{Q}^{r(l)} = \big[\omega_m - i\big(\beta \pm \tfrac{\Gamma_{R1}+\Gamma_{R2}}{2}\big)\big]\mathbf{I} + \begin{pmatrix} \pm i\frac{\Gamma_{R1}-\Gamma_{R2}}{2} & J_R \\ J_R & \mp i\frac{\Gamma_{R1}-\Gamma_{R2}}{2} \end{pmatrix}. \tag{S45}$$

The matrix elements are given by

$$\Gamma_{R1} = \frac{2\,\kappa_1\kappa_2\big[(\kappa_2 - 2\kappa_3) - 2\sqrt{\kappa_3(\kappa_1 - \kappa_2 + \kappa_3)}\big]}{\big(\kappa_1 - \kappa_2 + \kappa_3 + \sqrt{\kappa_3(\kappa_1 - \kappa_2 + \kappa_3)}\big)^2}, \tag{S46}$$

$$\Gamma_{R2} = -\frac{2\,\kappa_2\big[\kappa_1(\kappa_1 - \kappa_3) + \kappa_3(\kappa_2 - 2\kappa_3) - 2\kappa_3\sqrt{\kappa_3(\kappa_1 - \kappa_2 + \kappa_3)}\big]}{\big(\kappa_1 - \kappa_2 + \kappa_3 + \sqrt{\kappa_3(\kappa_1 - \kappa_2 + \kappa_3)}\big)^2}, \tag{S47}$$

$$J_R = -\frac{2\sqrt{\kappa_1\kappa_2}\,\big[(\kappa_1 + \kappa_3)(\kappa_2 - 2\kappa_3) - (\kappa_1 + 2\kappa_3)\sqrt{\kappa_3(\kappa_1 - \kappa_2 + \kappa_3)}\big]}{\big(\kappa_1 - \kappa_2 + \kappa_3 + \sqrt{\kappa_3(\kappa_1 - \kappa_2 + \kappa_3)}\big)^2}. \tag{S48}$$

The first term of Eq. (S45) represents a global frequency shift and uniform loss. The second term, being traceless with balanced gain and loss for the diagonal elements and symmetric coupling J_R for the off-diagonal elements, constitutes a canonical parity-time (PT)-symmetric structure. This

form makes the Hamiltonian $H_{\mathrm{RL}}^{r(l)}$ explicit and reveals the underlying mechanism for the emergence of exceptional points (EPs). The eigenfrequencies of $H_{\mathrm{RL}}^{r(l)}$ are given by

$$\omega_{\pm}^{r} = \omega_m - i\left(\beta + \frac{\Gamma_{R1}+\Gamma_{R2}}{2}\right) \pm \sqrt{J_R^2 - \left(\frac{\Gamma_{R1}-\Gamma_{R2}}{2}\right)^2} \tag{S49}$$

and

$$\omega_{\pm}^{l} = \omega_m - i\left(\beta - \frac{\Gamma_{R1}+\Gamma_{R2}}{2}\right) \pm \sqrt{J_R^2 - \left(\frac{\Gamma_{R1}-\Gamma_{R2}}{2}\right)^2}. \tag{S50}$$

Clearly, the eigenvalues in both directions share the same degeneracy condition

$$J_R = \frac{1}{2}|\Gamma_{R1} - \Gamma_{R2}|. \tag{S51}$$

Substituting the explicit forms of Γ_{R1}, Γ_{R2}, and J_R into Eqs. (S49) and (S50) yields the analytical dependence of the RL eigenfrequencies and reflection coefficient on the κ_1, κ_2, and κ_3. The resulting expressions are

$$R^{r(l)} = -i(\kappa_1 - \kappa_2 + \kappa_3)\frac{(\omega - \omega_{+}^{r,l})(\omega - \omega_{-}^{r,l})}{\det(\omega\mathbf{I} - H_{\mathrm{eff}}^{\text{anti-Bragg}})}, \tag{S52}$$

where

$$\omega_{\pm}^{r} = \omega_m - i\beta + \frac{i\kappa_2(\kappa_1 - \kappa_3) \pm \sqrt{\kappa_2(4\kappa_1 - \kappa_2)(\kappa_1 + \kappa_3)\left(\kappa_3 - \frac{\kappa_1\kappa_2}{4\kappa_1-\kappa_2}\right)}}{\kappa_1 - \kappa_2 + \kappa_3}, \tag{S53a}$$

$$\omega_{\pm}^{l} = \omega_m - i\beta + \frac{-i\kappa_2(\kappa_1 - \kappa_3) \pm \sqrt{\kappa_2(4\kappa_1 - \kappa_2)(\kappa_1 + \kappa_3)\left(\kappa_3 - \frac{\kappa_1\kappa_2}{4\kappa_1-\kappa_2}\right)}}{\kappa_1 - \kappa_2 + \kappa_3}. \tag{S53b}$$

When $\kappa_1 > \kappa_2 > \kappa_3$, equations (S53a) and (S53b) show that the degeneracy condition in both directions as

$$\kappa_3 = \frac{\kappa_1\kappa_2}{4\kappa_1 - \kappa_2}. \tag{S54}$$

The eigenvalues in two directions become different when $\kappa_1 \neq \kappa_3$, leading to the asymmetric reflection spectra. Since the experimental probing frequency ω is purely real, only RL states with real eigenvalues ($\mathrm{Im}(\omega_{\pm}^{r(l)}) = 0$) can be observed in the spectra measurement. However, the RL states in both direction cannot be observed simultaneously since $\mathrm{Im}(\omega_{\pm}^{r}) \neq \mathrm{Im}(\omega_{\pm}^{l})$ when $\kappa_1 \neq \kappa_3$. Because κ_1 is much larger than κ_3 in our experiment, this is the fundamental reason we can observe the unidirectional RL state. Thus, the emergence of the unidirectional RL EP corresponds to the pure real and dengerated RL eigenvalues in one direction. By substituting these two conditions

into the RL Hamiltonians of two directions, we find that H^r_{RL} satisfies the PT symmetry, while the H^l_{RL} does not. This difference is the underlying mechanism enabling the construction of the unidirectional RL EP in our system. The above mathematical procedure yields the same solution as setting the numerator of the reflection coefficient to zero in the main text, but it clarifies the origin of this unidirectional second-order EP.

S7. The distinction between spectral in linear and decibel (dB) scales of reflection

In Supplementary Fig. S3, we present the evolution of the reflection spectrum as κ_3 varies while κ_1 and κ_2 are kept fixed. Linear-scale representations are shown in Supplementary Figs. S3A and S3AB, with their corresponding dB-scale counterparts provided in Figs. S3C and S3D. At the unidirectional RL EP, the critical value is derived as $\kappa_3 = \frac{\kappa_1\kappa_2}{4\kappa_1-\kappa_2}$.

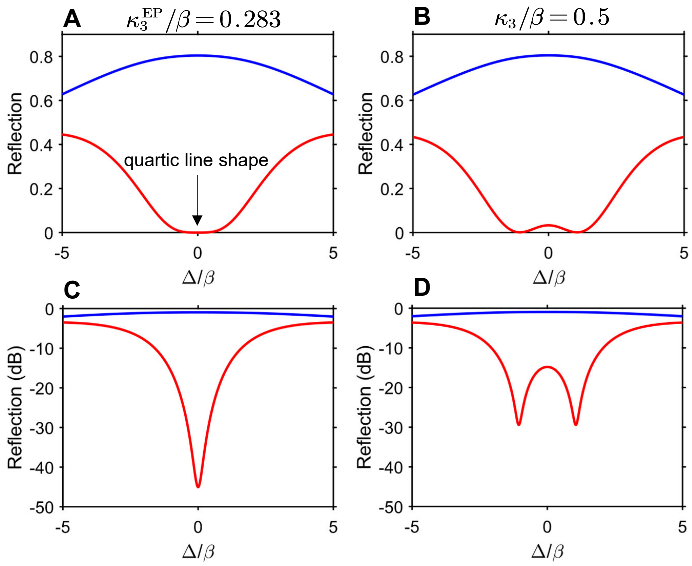

Figure S3: **Reflection spectra at and near the unidirectional RL EP.** (**A**) and (**C**) display the reflection spectra under RL EP conditions ($\kappa_3/\beta = 0.283$) on linear and dB scales, respectively. (**B**) and (**D**) show the spectra for $\kappa_3/\beta = 0.5$ on linear and dB scales, respectively. These results are acquired when $\kappa_1/\beta = 9$ and $\kappa_2/\beta = 1.1$.

The characteristic quartic lineshape (*19, 21*) is clearly resolved in Fig. S3A at the RL EP. The corresponding sharp dip on the dB scale in Fig. S3C further confirms the degeneracy of the reflectionless states. However, deviation from EP conditions eliminates the quartic dependence in

Supplementary Fig. S3B, thereby its unidirectional bandwidth is significantly reduced.

S8. Cavity Magnonic System with Magnonic Mirrors

Given that the first and third spin ensembles are separated by a distance of $\lambda_m/2$, the effective non-Hermitian Hamiltonian $H_{\text{eff}}^{\text{anti-Bragg}}$ can be transformed from the single-excitation basis $\{\sigma_i^\dagger|g_1g_2g_3\rangle\}$ to a new orthonormal basis $\{|B\rangle, |D\rangle, \sigma_2^\dagger|g_1g_2g_3\rangle\}$, where the bright and dark states are defined as

$$|B\rangle = \frac{\sqrt{\kappa_3}|egg\rangle - \sqrt{\kappa_1}|gge\rangle}{\sqrt{\kappa_1+\kappa_3}}, \tag{S55a}$$

$$|D\rangle = \frac{\sqrt{\kappa_3}|egg\rangle + \sqrt{\kappa_1}|gge\rangle}{\sqrt{\kappa_1+\kappa_3}}. \tag{S55b}$$

A unitary transformation matrix is applied as follows

$$U = \begin{pmatrix} \frac{\sqrt{\kappa_3}}{\sqrt{\kappa_1+\kappa_3}} & 0 & \frac{\sqrt{\kappa_1}}{\sqrt{\kappa_1+\kappa_3}} \\ 0 & 1 & 0 \\ \frac{\sqrt{\kappa_1}}{\sqrt{\kappa_1+\kappa_3}} & 0 & \frac{-\sqrt{\kappa_3}}{\sqrt{\kappa_1+\kappa_3}} \end{pmatrix}, \tag{S56}$$

which transforms the effective Hamiltonian to

$$H_c = U^\dagger H_{\text{eff}}^{\text{anti-Bragg}} U = \begin{pmatrix} -i\Gamma_D & J_D & 0 \\ J_D & -i\gamma & J_B \\ 0 & J_B & -i\Gamma_B \end{pmatrix}. \tag{S57}$$

In this representation, the system is analogous to a cavity magnonic system, where the second magnon mode couples to two cavity modes defined by the bright and dark states, while the first and third spin ensemble, separated by $\lambda_m/2$, function as a pair of magnonic mirrors. Here, $J_{B(D)}$ are the coherent coupling strengths between the second magnon mode and the bright (dark) states, and the decay rates $\Gamma_{B(D)}$ correspond to their loss. The external decay of the second spin ensemble is denoted as $\gamma = \beta + \kappa_2$. These parameters are given by

$$J_D = \frac{2\sqrt{\kappa_1\kappa_2\kappa_3}}{\sqrt{\kappa_1+\kappa_3}}, \qquad J_B = \frac{\sqrt{\kappa_2}(\kappa_1-\kappa_3)}{\sqrt{\kappa_1+\kappa_3}},$$

$$\Gamma_D = \beta, \qquad \Gamma_B = \beta + \kappa_1 + \kappa_3.$$

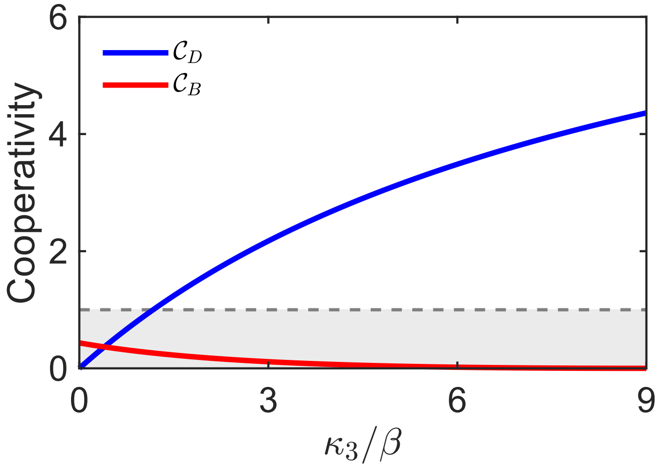

Figure S4: **Cooperativity of the cavity magnonic system.** Parameters: $\kappa_1/\beta = 9.1$, $\kappa_2/\beta = 0.93$. The gray region indicates $C < 1$.

S9. Influence of the bright state on reflection spectra

In the symmetric case with $\kappa_1 = \kappa_3$, the coupling strength $J_B = 0$, implying that the middle magnon mode only couples to the dark state (*54*). In this work, we focus on the asymmetric case with $\kappa_1 \gg \kappa_3$. To quantify the effective coupling regime, we introduce the cooperativity parameters for dark and bright state coupling (*31*)

$$C_D = \frac{J_D^2}{\gamma\Gamma_D} = \frac{2\kappa_1\kappa_2\kappa_3}{\beta(\beta+\kappa_2)(\kappa_1+\kappa_3)}, \tag{S58a}$$

$$C_B = \frac{J_B^2}{\gamma\Gamma_B} = \frac{\kappa_2(\kappa_1-\kappa_3)^2}{(\beta+\kappa_2)(\beta+\kappa_1+\kappa_3)(\kappa_1+\kappa_3)}. \tag{S58b}$$

It can be shown that $C_B \leq 1$ for all non-negative values of κ_1, κ_2, κ_3, and β. Since J_B is naturally smaller than J_D and Γ_B is much larger than Γ_D, C_B is almost always smaller than C_D over the range of κ_3 (Fig. S4). Therefore, the dark state and middle magnon mode exhibit pronounced coherent behaviors, while the bright state behaves more like a dissipative reservoir rather than supporting coherent coupling. More importantly, C_D can be tuned to significantly exceed unity, allowing the strong coupling between the middle magnon mode and the dark state, as illustrated in Fig. S4. To further assess the influence of asymmetry on our analysis, we plot the reflection coefficients for both propagation directions, along with the contrast ratio $C = |(|R^r|^2 - |R^l|)|^2/|(R^r|^2 + |R^l|^2)|$, versus the coupling strength κ_3 and the detuning Δ in Supplementary Fig. S5. We observe that the bright state

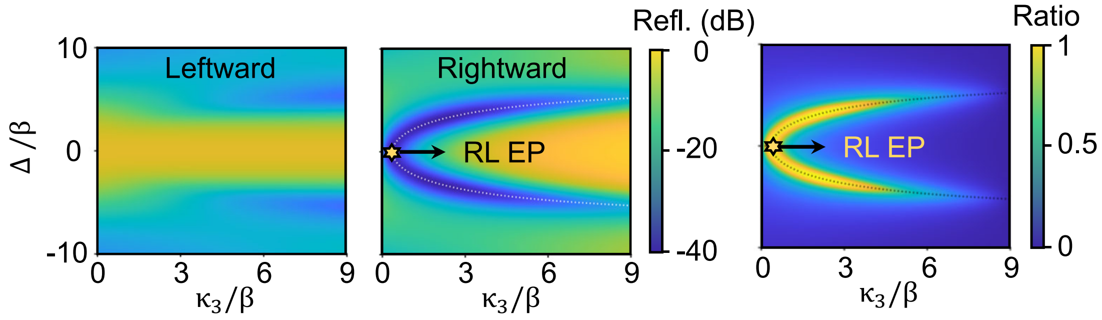

Figure S5: **Mapping of asymmetric reflection spectra.** Leftward reflection, rightward reflection, and their contrast ratio versus κ_3. Dashed curves show the corresponding RL eigenfrequencies of the magnonic mirror array system. Parameters: $\kappa_1/\beta = 9$, $\kappa_2/\beta = 0.93$.

consistently manifests in both reflection spectra as a central, broad and high-reflectivity plateau that is insensitive to asymmetry between κ_1 and κ_3. This follows from its broad linewidth and strong coupling to the probe channel. Thus, the distinct RL dips observed for right incidence just signifies the coupling regimes transition of the equivalent magnonic mirror cavity system composed of the dark state and second magnon mode. The above results illustrate that the bright state can be resonably neglected in the reflection spectra measurement. These unidirectional RL states provide a unique opportunity to directly observe magnonic mirror array (MMA) polariton behaviors via waveguide transmission in a single direction. This directionality persists until the system regains inversion symmetry, as illustrated in Supplementary Fig. S5 when $\kappa_1/\beta = \kappa_3/\beta \approx 9$.

S10. Fitting of the reflectionless state eigenvalues

Despite the quartic dip observed in the spectrum, the definitive verification of the RL EP relies on the degeneracy of the RL-state eigenvalues. These eigenvalues are extracted by fitting the reflection spectra measured under the condition that all three magnon modes are brought into resonance. To provide a clearer illustration of the fitting procedure, we additionally present two representative reflection spectra, distinct from those shown in Figs. 4G and 4I of the main text, corresponding respectively to the regimes before and far beyond the RL EP (Figs. S6A and S6B). In the former case ($\kappa_3/\beta = 0.173$, Fig. S6A), the real parts of $\omega_{\mathrm{RL}}^{l,\pm}$ coalesce while the imaginary parts remain distinct. As a result, the S_{11} spectrum features a single Lorentzian dip with near-zero reflection, rather than the characteristic quartic reflectionless dip that emerges at the RL EP. In the latter

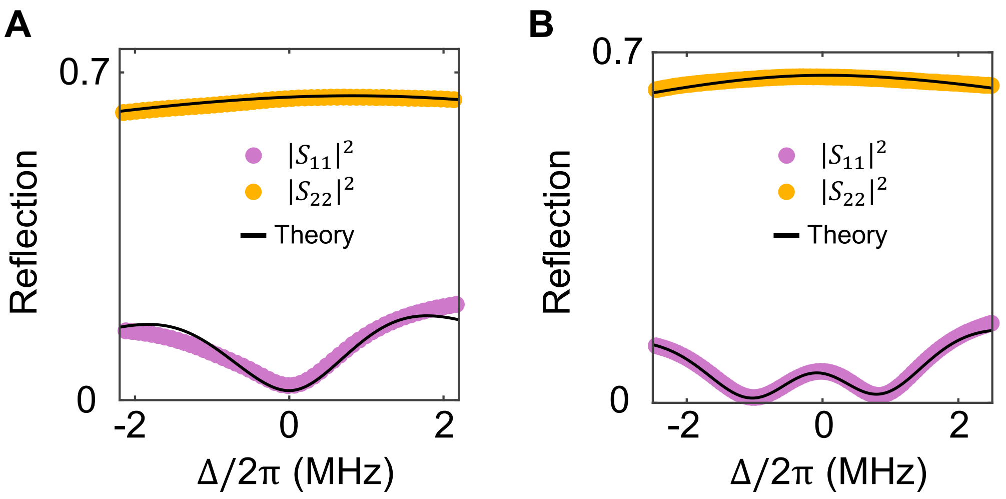

Figure S6: **Fitting of the RL-state eigenvalues.** (**A** and **B**) Representative reflection spectra measured from both waveguide ports (port 1 and port 2) at $\kappa_3/\beta = 0.173$ (A) and $\kappa_3/\beta = 0.73$ (B). The circular points indicate the experimental data, and the solid curves show the corresponding theoretical fits.

case ($\kappa_3/\beta = 0.73$, Fig. S6B), the real parts split more profoundly than in Fig. 4I, whereas the imaginary parts merge. Consequently, the S_{11} spectrum displays two well-separated but identical Lorentzian reflection dips. As S_{22} remains a broad reflection peak throughout the variation of κ_3, the corresponding RL-state eigenvalues $\omega_{\mathrm{RL}}^{r,\pm}$ cannot capture the resonance characteristics of the spectrum (*16*). This behavior is fully consistent with our theoretical analysis in Supplementary Section S9, which predicts that the RL states and the RL EP emerge unidirectionally in this system. The numerical fitting therefore provides further confirmation of the appearance of unidirectional RL EP.

S11. Device Photo

Supplementary Fig. S7 shows a device photograph that clearly displays the magnonic mirror array used in our experiment.

S12. Phase-Dependent Reflection Asymmetry

To investigate the influence of the phase on reflection asymmetry, we consider two magnon modes cooperatively coupled to the waveguide. As schematically illustrated in Supplementary Fig. S8A,

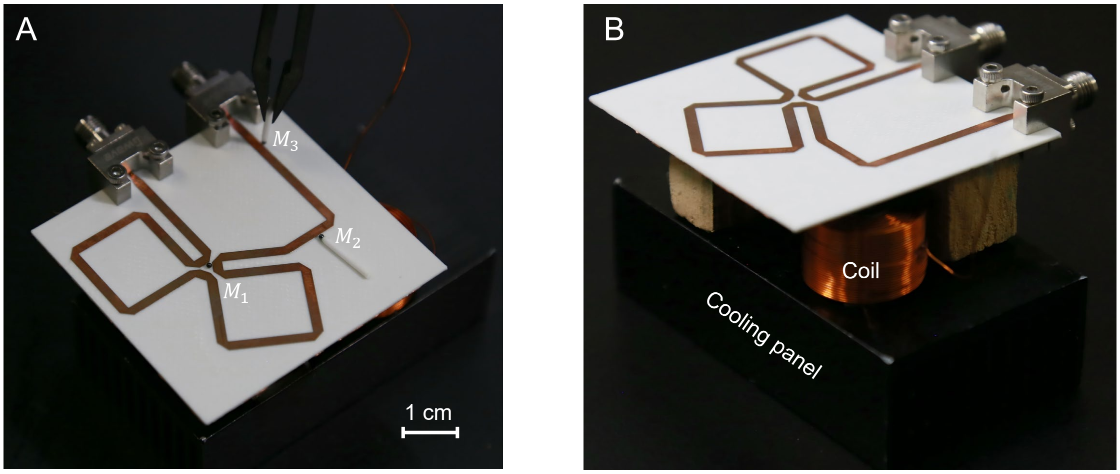

Figure S7: **Device photo.** (**A**) Top view photograph of the experimental setup, where the overall chip area is 540 mm × 520 mm. (**B**) Side view photograph of the experimental setup. The coil applies a local magnetic field at M_2 and a panel attached at the bottom is used to cool the coil.

the relative phase of two YIG spheres is $\varphi = kd$, where $d = x_2 - x_1$ is the separation. Under this configuration, the Hamiltonian H_{eff} and coupling vector V take the following forms

$$H_{\text{eff}} = \begin{pmatrix} \omega_m - i(\beta + \kappa_1) & -i\sqrt{\kappa_1\kappa_2}\, e^{i\varphi} \\ -i\sqrt{\kappa_1\kappa_2}\, e^{i\varphi} & \omega_m - i(\beta + \kappa_2) \end{pmatrix}, \qquad \mathbf{V} = \begin{pmatrix} \sqrt{\kappa_1} \\ \sqrt{\kappa_2}\, e^{i\varphi} \end{pmatrix}. \tag{S59}$$

By substituting (S59) into (S8b), the rightward reflection coefficient can be deduced as

$$R^r = \frac{\kappa_2 e^{i2\varphi}\left[\beta - \kappa_1 - i(\omega - \omega_m)\right] + \kappa_1\left[\beta + \kappa_2 - i(\omega - \omega_m)\right]}{\kappa_1\kappa_2 e^{i2\varphi} - \left[\beta + \kappa_1 - i(\omega - \omega_m)\right]\left[\beta + \kappa_2 - i(\omega - \omega_m)\right]}. \tag{S60}$$

The corresponding leftward reflection coefficient R^l follows by swapping the subscripts $1 \leftrightarrow 2$. Thus, the asymmetry of the reflection is defined as

$$A = |R^r|^2 - |R^l|^2 = \frac{8\beta\kappa_1\kappa_2\left(\kappa_1 - \kappa_2\right)\sin^2\varphi}{\left|\left(\beta - i(\omega - \omega_m) + \kappa_1\right)\left(\beta - i(\omega - \omega_m) + \kappa_2\right)e^{2i\varphi} - \kappa_1\kappa_2\right|^2}, \tag{S61}$$

which directly reveals how the phase influences the degree of reflection asymmetry. As shown by the numerical results in Supplementary Fig. S8B, the reflection spectra evolve continuously from asymmetric to symmetric as φ varies from $\pi/2$ to π, confirming that the transition changes gradually rather than abruptly. Experimentally, the phase was tuned by varying the distance between the two YIG spheres via step motors. By fitting the asymmetry between the measured rightward and leftward reflection spectra, we extracted the position-phase relation displayed as the dots

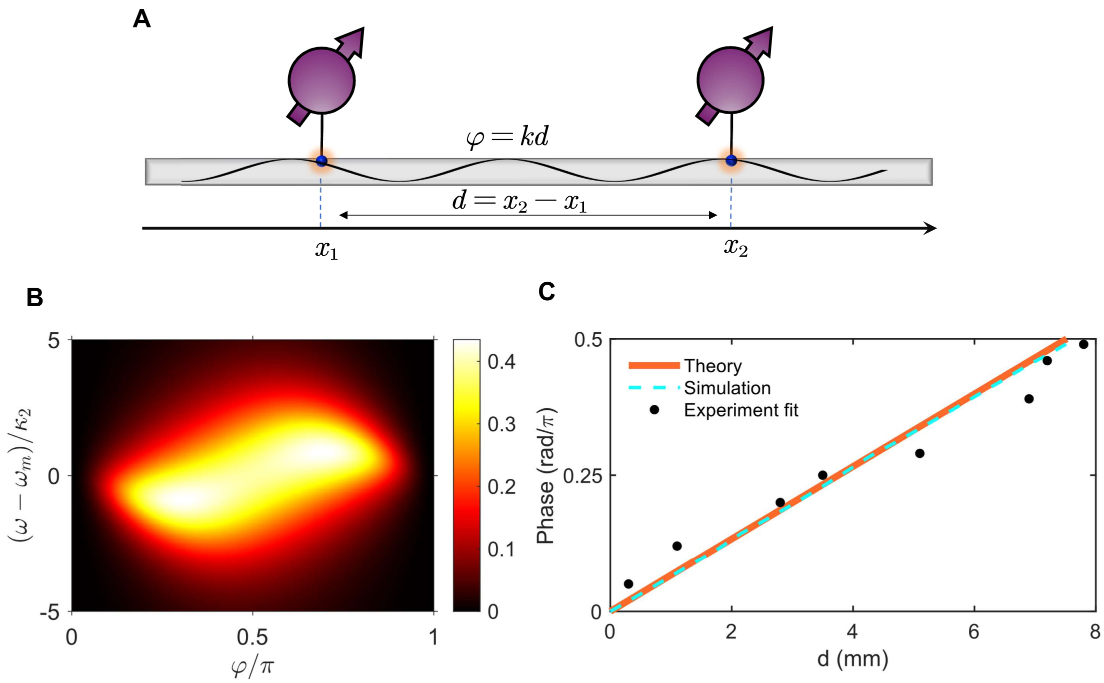

Figure S8: **Phase-dependent reflection asymmetry in the waveguide magnonic system. (A)** Schematic of two YIG spheres coupled to the microstrip with separation d, introducing a phase $\varphi = kd$. **(B)** Calculated reflection asymmetry $A = |R^r|^2 - |R^l|^2$ as a function of phase φ and frequency detuning $(\omega - \omega_m)$. Parameters: $\kappa_1/\kappa_2 = 3$, $\beta/\kappa_2 = 1$. **(C)** Experimentally extracted relation between position and phase. Dots, the solid and dashed lines represent experimental data, theoretical fit and simulation result, respectively.

in Supplementary Fig. S8C. Since the effective wave number of the microstrip at the operating frequency $\omega/2\pi \approx 5.8$ GHz is constant ($k_{\text{eff}} \approx 209$ m^{-1}), the position–phase dependence agrees well with a linear fit (solid line in Supplementary Fig. S8C). This result is further validated by the CST simulations (dashed line in Supplementary Fig. S8C).

REFERENCES

1. L. Feng, Y. L. Xu, W. S. Fegadolli, M. H. Lu, J. E. Oliveira, V. R. Almeida, Y. F. Chen, A. Scherer, Experimental demonstration of a unidirectional reflectionless parity-time metamaterial at optical frequencies. *Nat. Mater.* **12**, 108–113 (2013).

2. Z. Lin, H. Ramezani, T. Eichelkraut, T. Kottos, H. Cao, D. N. Christodoulides, Unidirectional invisibility induced by PT-symmetric periodic structures. *Phys. Rev. Lett.* **106**, 213901 (2011).

3. S. Longhi, Invisibility in PT-symmetric complex crystals. *J. Phys.s A Math. Theor.* **44**, 485302 (2011).

4. H. Jones, Analytic results for a PT-symmetric optical structure. *J. Phys. A Math. Theor.* **45**, 135306 (2012).

5. S. Soleymani, Q. Zhong, M. Mokim, S. Rotter, R. El-Ganainy, Ş. K. Özdemir, Chiral and degenerate perfect absorption on exceptional surfaces. *Nat. Commun.* **13**, 599 (2022).

6. L. Feng, X. Zhu, S. Yang, H. Zhu, P. Zhang, X. Yin, Y. Wang, X. Zhang, Demonstration of a large-scale optical exceptional point structure. *Opt. Express* **22**, 1760 (2014).

7. Y. Huang, Y. Shen, C. Min, S. Fan, G. Veronis, Unidirectional reflectionless light propagation at exceptional points. *Nanophotonics* **6**, 977–996 (2017).

8. X. Yin, X. Zhang, Unidirectional light propagation at exceptional points. *Nat. Mater.* **12**, 175–177 (2013).

9. Y. Jia, Y. Yan, S. V. Kesava, E. D. Gomez, N. C. Giebink, Passive parity-time symmetry in organic thin film waveguides. *ACS Photonics* **2**, 319–325 (2015).

10. H. Qin, Z. Yang, P.-S. Huang, X. Mu, S.-H. Huang, Y. Shi, W. Zhao, B. Li, J. Zhou, J. Zúñiga-Pérez, P. Genevet, P. C. Wu, Q. Song, Sphere of arbitrarily polarized exceptional points with a single planar metasurface. *Nat. Commun.* **16**, 2656 (2025).

11. M. Lawrence, N. Xu, X. Zhang, L. Cong, J. Han, W. Zhang, S. Zhang, Manifestation of *PT* symmetry breaking in polarization space with terahertz metasurfaces. *Phys. Rev. Lett.* **113**, 093901 (2014).

12. Z. Yang, P.-S. Huang, Y.-T. Lin, H. Qin, J. Zúñiga-Pérez, Y. Shi, Z. Wang, X. Cheng, M.-C. Tang, S. Han, B. Kanté, B. Li, P. C. Wu, P. Genevet, Q. Song, Creating pairs of exceptional points for arbitrary polarization control: Asymmetric vectorial wavefront modulation. *Nat. Commun.* **15**, 232 (2024).

13. J.-H. Wu, M. Artoni, G. C. La Rocca, Non-Hermitian degeneracies and unidirectional reflectionless atomic lattices. *Phys. Rev. Lett.* **113**, 123004 (2014).

14. J. Qian, C. H. Meng, J. W. Rao, Z. J. Rao, Z. An, Y. Gui, C.-M. Hu, Non-Hermitian control between absorption and transparency in perfect zero-reflection magnonics. *Nat. Commun.* **14**, 3437 (2023).

15. J. W. Rao, P. C. Xu, Y. S. Gui, Y. P. Wang, Y. Yang, B. Yao, J. Dietrich, G. E. Bridges, X. L. Fan, D. S. Xue, C.-M. Hu, Interferometric control of magnon-induced nearly perfect absorption in cavity magnonics. *Nat. Commun.* **12**, 1933 (2021).

16. Y. Han, C. Meng, H. Pan, J. Qian, Z. Rao, L. Zhu, Y. Gui, C. M. Hu, Z. An, Bound chiral magnonic polariton states for ideal microwave isolation. *Sci. Adv.* **9**, eadg4730 (2023).

17. Z. Rao, C. Meng, Y. Han, L. Zhu, K. Ding, Z. An, Braiding reflectionless states in non-Hermitian magnonics. *Nat. Phys.* **20**, 1904–1911 (2024).

18. C. Ferise, P. Del Hougne, S. Félix, V. Pagneux, M. Davy, Exceptional points of PT-symmetric reflectionless states in complex scattering systems. *Phys. Rev. Lett.* **128**, 203904 (2022).

19. W. R. Sweeney, C. W. Hsu, S. Rotter, A. D. Stone, Perfectly absorbing exceptional points and chiral absorbers. *Phys. Rev. Lett.* **122**, 093901 (2019).

20. W. R. Sweeney, C. W. Hsu, A. D. Stone, Theory of reflectionless scattering modes. *Phys. Rev. A* **102**, 063511 (2020).

21. Y. Yang, Y.-P. Wang, J. W. Rao, Y. S. Gui, B. M. Yao, W. Lu, C.-M. Hu, Unconventional singularity in anti-parity-time symmetric cavity magnonics. *Phys. Rev. Lett.* **125**, 147202 (2020).

22. H. Hörner, L. Wild, Y. Slobodkin, G. Weinberg, O. Katz, S. Rotter, Coherent perfect absorption of arbitrary wavefronts at an exceptional point. *Phys. Rev. Lett.* **133**, 173801 (2024).

23. W. Chen, Ş. Kaya Özdemir, G. Zhao, J. Wiersig, L. Yang, Exceptional points enhance sensing in an optical microcavity. *Nature* **548**, 192–196 (2017).

24. H. Hodaei, A. U. Hassan, S. Wittek, H. Garcia-Gracia, R. el-Ganainy, D. N. Christodoulides, M. Khajavikhan, Enhanced sensitivity at higher-order exceptional points. *Nature* **548**, 187–191 (2017).

25. Z. Xiao, H. Li, T. Kottos, A. Alù, Enhanced sensing and nondegraded thermal noise performance based on PT-symmetric electronic circuits with a sixth-order exceptional point. *Phys. Rev. Lett.* **123**, 213901 (2019).

26. J. Wiersig, Prospects and fundamental limits in exceptional point-based sensing. *Nat. Commun.* **11**, 2454 (2020).

27. H. Wang, Y.-H. Lai, Z. Yuan, M.-G. Suh, K. Vahala, Petermann-factor sensitivity limit near an exceptional point in a Brillouin ring laser gyroscope. *Nat. Commun.* **11**, 1610 (2020).

28. R. Kononchuk, J. Cai, F. Ellis, R. Thevamaran, T. Kottos, Exceptional-point-based accelerometers with enhanced signal-to-noise ratio. *Nature* **607**, 697–702 (2022).

29. J. Wiersig, Enhancing the sensitivity of frequency and energy splitting detection by using exceptional points: Application to microcavity sensors for single-particle detection. *Phys. Rev. Lett.* **112**, 203901 (2014).

30. D. Roy, C. Wilson, O. Firstenberg, *Colloquium*: Strongly interacting photons in one-dimensional continuum. *Rev. Mod. Phys.* **89**, 021001 (2017).

31. A. S. Sheremet, M. I. Petrov, I. V. Iorsh, A. V. Poshakinskiy, A. N. Poddubny, Waveguide quantum electrodynamics: Collective radiance and photon-photon correlations. *Rev. Mod. Phys.* **95**, 015002 (2023).

32. A. F. Van Loo, A. Fedorov, K. Lalumière, B. C. Sanders, A. Blais, A. Wallraff, Photon-mediated interactions between distant artificial atoms. *Science* **342**, 1494–1496 (2013).

33. N. V. Corzo, J. Raskop, A. Chandra, A. S. Sheremet, B. Gouraud, J. Laurat, Waveguide-coupled single collective excitation of atomic arrays. *Nature* **566**, 359–362 (2019).

34. D. Mukhopadhyay, G. S. Agarwal, Multiple Fano interferences due to waveguide-mediated phase coupling between atoms. *Phys. Rev. A* **100**, 013812 (2019).

35. Z.-Q. Wang, Y.-P. Wang, J. Yao, R.-C. Shen, W.-J. Wu, J. Qian, J. Li, S.-Y. Zhu, J. Q. You, Giant spin ensembles in waveguide magnonics. *Nat. Commun.* **13**, 7580 (2022).

36. A. Frisk Kockum, P. Delsing, G. Johansson, Designing frequency-dependent relaxation rates and Lamb shifts for a giant artificial atom. *Phys. Rev. A* **90**, 013837 (2014).

37. A. F. Kockum, G. Johansson, F. Nori, Decoherence-free interaction between giant atoms in waveguide quantum electrodynamics. *Phys. Rev. Lett.* **120**, 140404 (2018).

38. L. Guo, A. Grimsmo, A. F. Kockum, M. Pletyukhov, G. Johansson, Giant acoustic atom: A single quantum system with a deterministic time delay. *Phys. Rev. A* **95**, 053821 (2017).

39. A. González-Tudela, C. S. Muñoz, J. Cirac, Engineering and harnessing giant atoms in high-dimensional baths: A proposal for implementation with cold atoms. *Phys. Rev. Lett.* **122**, 203603 (2019).

40. B. Kannan, M. J. Ruckriegel, D. L. Campbell, A. Frisk Kockum, J. Braumüller, D. K. Kim, M. Kjaergaard, P. Krantz, A. Melville, B. M. Niedzielski, A. Vepsäläinen, R. Winik, J. L. Yoder, F. Nori, T. P. Orlando, S. Gustavsson, W. D. Oliver, Waveguide quantum electrodynamics with superconducting artificial giant atoms. *Nature* **583**, 775–779 (2020).

41. G. Andersson, B. Suri, L. Guo, T. Aref, P. Delsing, Non-exponential decay of a giant artificial atom. *Nat. Phys.* **15**, 1123–1127 (2019).

42. A. Frisk Kockum, *Quantum Optics with Giant Atoms–the First Five Years* (Springer Singapore, 2021), vol. 33.

43. X. Wang, T. Liu, A. F. Kockum, H.-R. Li, F. Nori, Tunable chiral bound states with giant atoms. *Phys. Rev. Lett.* **126**, 043602 (2021).

44. Y. P. Peng, W. Z. Jia, Single-photon scattering from a chain of giant atoms coupled to a one-dimensional waveguide. *Phys. Rev. A* **108**, 043709 (2023).

45. A. M. Vadiraj, A. Ask, T. G. McConkey, I. Nsanzineza, C. W. S. Chang, A. F. Kockum, C. M. Wilson, Engineering the level structure of a giant artificial atom in waveguide quantum electrodynamics. *Phys. Rev. A* **103**, 023710 (2021).

46. F. Roccati, D. Cilluffo, Controlling Markovianity with chiral giant atoms. *Phys. Rev. Lett.* **133**, 063603 (2024).

47. L. Du, Y. Zhang, J.-H. Wu, A. F. Kockum, Y. Li, Giant atoms in a synthetic frequency dimension. *Phys. Rev. Lett.* **128**, 223602 (2022).

48. M.-A. Miri, A. Alù, Exceptional points in optics and photonics. *Science* **363**, eaar7709 (2019).

49. S. K. Ozdemir, S. Rotter, F. Nori, L. Yang, Parity–time symmetry and exceptional points in photonics. *Nat. Mater.* **18**, 783–798 (2019).

50. R. El-Ganainy, K. G. Makris, M. Khajavikhan, Z. H. Musslimani, S. Rotter, D. N. Christodoulides, Non-Hermitian physics and PT symmetry. *Nat. Phys.* **14**, 11–19 (2018).

51. R. H. Dicke, Coherence in spontaneous radiation processes. *Phys. Rev.* **93**, 99–110 (1954).

52. A. Tiranov, V. Angelopoulou, C. van Diepen, B. Schrinski, O. A. D. Sandberg, Y. Wang, L. Midolo, S. Scholz, A. D. Wieck, A. Ludwig, A. S. Sørensen, P. Lodahl, Collective super- and subradiant dynamics between distant optical quantum emitters. *Science* **379**, 389–393 (2023).

53. M. Zanner, T. Orell, C. M. F. Schneider, R. Albert, S. Oleschko, M. L. Juan, M. Silveri, G. Kirchmair, Coherent control of a multi-qubit dark state in waveguide quantum electrodynamics. *Nat. Phys.* **18**, 538–543 (2022).

54. M. Mirhosseini, E. Kim, X. Zhang, A. Sipahigil, P. B. Dieterle, A. J. Keller, A. Asenjo-Garcia, D. E. Chang, O. Painter, Cavity quantum electrodynamics with atom-like mirrors. *Nature* **569**, 692–697 (2019).

55. D. Zhang, X.-Q. Luo, Y.-P. Wang, T.-F. Li, J. Q. You, Observation of the exceptional point in cavity magnon-polaritons. *Nat. Commun.* **8**, 1368 (2017).

56. J. Rao, C. Y. Wang, B. Yao, Z. J. Chen, K. X. Zhao, W. Lu, Meterscale strong coupling between magnons and photons. *Phys. Rev. Lett.* **131**, 106702 (2023).

57. J. Xu, C. Zhong, S. Zhuang, C. Qian, Y. Jiang, A. Pishehvar, X. Han, D. Jin, J. M. Jornet, B. Zhen, J. Hu, L. Jiang, X. Zhang, Slow-wave hybrid magnonics. *Phys. Rev. Lett.* **132**, 116701 (2024).

58. B. Yao, Y. S. Gui, J. W. Rao, Y. H. Zhang, W. Lu, C.-M. Hu, Coherent microwave emission of gain-driven polaritons. *Phys. Rev. Lett.* **130**, 146702 (2023).

59. C. Wang, J. Rao, Z. Chen, K. Zhao, L. Sun, B. Yao, T. Yu, Y.-P. Wang, W. Lu, Enhancement of magnonic frequency combs by exceptional points. *Nat. Phys.* **20**, 1139–1144 (2024).

60. Y. Cao, P. Yan, Exceptional magnetic sensitivity of PT-symmetric cavity magnon polaritons. *Phys. Rev. B* **99**, 214415 (2019).

61. J. Qian, J. Li, S.-Y. Zhu, J. Q. You, Y.-P. Wang, Probing *PT*-symmetry breaking of non-Hermitian topological photonic states via strong photon-magnon coupling. *Phys. Rev. Lett.* **132**, 156901 (2024).

62. G.-T. Xu, M. Zhang, Y. Wang, Z. Shen, G.-C. Guo, C.-H. Dong, Magnonic frequency comb in the magnomechanical resonator. *Phys. Rev. Lett.* **131**, 243601 (2023).

63. S. P. Wolski, D. Lachance-Quirion, Y. Tabuchi, S. Kono, A. Noguchi, K. Usami, Y. Nakamura, Dissipation-based quantum sensing of magnons with a superconducting qubit. *Phys. Rev. Lett.* **125**, 117701 (2020).

64. V. L. Grigoryan, K. Shen, K. Xia, Synchronized spin-photon coupling in a microwave cavity. *Phys. Rev. B* **98**, 024406 (2018).

65. B. Zare Rameshti, S. V. Kusminskiy, J. A. Haigh, K. Usami, D. Lachance-Quirion, Y. Nakamura, C.-M. Hu, H. X. Tang, G. E. W. Bauer, Y. M. Blanter, Cavity magnonics. *Phys. Rep.* **979**, 1–61 (2022).

66. H. Huebl, C. W. Zollitsch, J. Lotze, F. Hocke, M. Greifenstein, A. Marx, R. Gross, S. T. Goennenwein, High cooperativity in coupled microwave resonator ferrimagnetic insulator hybrids. *Phys. Rev. Lett.* **111**, 127003 (2013).

67. R.-C. Shen, Y.-P. Wang, J. Li, S.-Y. Zhu, G. S. Agarwal, J. Q. You, Long-time memory and ternary logic gate using a multistable cavity magnonic system. *Phys. Rev. Lett.* **127**, 183202 (2021).

68. R.-C. Shen, J. Li, Z.-Y. Fan, Y.-P. Wang, J. Q. You, Mechanical bistability in Kerr-modified cavity magnomechanics. *Phys. Rev. Lett.* **129**, 123601 (2022).

69. Y. Li, T. Polakovic, Y.-L. Wang, J. Xu, S. Lendinez, Z. Zhang, J. Ding, T. Khaire, H. Saglam, R. Divan, J. Pearson, W.-K. Kwok, Z. Xiao, V. Novosad, A. Hoffmann, W. Zhang, Strong coupling between magnons and microwave photons in on-chip ferromagnet-superconductor thin-film devices. *Phys. Rev. Lett.* **123**, 107701 (2019).

70. H. Yuan, Y. Cao, A. Kamra, R. A. Duine, P. Yan, Quantum magnonics: When magnon spintronics meets quantum information science. *Phys. Rep.* **965**, 1–74 (2022).

71. Y. Tabuchi, S. Ishino, T. Ishikawa, R. Yamazaki, K. Usami, Y. Nakamura, Hybridizing ferromagnetic magnons and microwave photons in the quantum limit. *Phys. Rev. Lett.* **113**, 083603 (2014).

72. X. Zhang, C.-L. Zou, L. Jiang, H. X. Tang, Strongly coupled magnons and cavity microwave photons. *Phys. Rev. Lett.* **113**, 156401 (2014).

73. Y. Tabuchi, S. Ishino, A. Noguchi, T. Ishikawa, R. Yamazaki, K. Usami, Y. Nakamura, Coherent coupling between a ferromagnetic magnon and a superconducting qubit. *Science* **349**, 405–408 (2015).

74. Y.-P. Wang, G.-Q. Zhang, D. Zhang, T.-F. Li, C.-M. Hu, J. Q. You, Bistability of cavity magnon polaritons. *Phys. Rev. Lett.* **120**, 057202 (2018).

75. Y.-P. Wang, J. W. Rao, Y. Yang, P.-C. Xu, Y. S. Gui, B. M. Yao, J. Q. You, C.-M. Hu, Nonreciprocity and unidirectional invisibility in cavity magnonics. *Phys. Rev. Lett.* **123**, 127202 (2019).

76. M. Harder, Y. Yang, B. M. Yao, C. H. Yu, J. W. Rao, Y. S. Gui, R. L. Stamps, C.-M. Hu, Level attraction due to dissipative magnon-photon coupling. *Phys. Rev. Lett.* **121**, 137203 (2018).

77. T. Yu, Y.-X. Zhang, S. Sharma, X. Zhang, Y. M. Blanter, G. E. W. Bauer, Magnon accumulation in chirally coupled magnets. *Phys. Rev. Lett.* **124**, 107202 (2020).

78. D. Xu, X.-K. Gu, H.-K. Li, Y.-C. Weng, Y.-P. Wang, J. Li, H. Wang, S.-Y. Zhu, J. Q. You, Quantum control of a single magnon in a macroscopic spin system. *Phys. Rev. Lett.* **130**, 193603 (2023).

79. D. Lachance-Quirion, S. P. Wolski, Y. Tabuchi, S. Kono, K. Usami, Y. Nakamura, Entanglement-based single-shot detection of a single magnon with a superconducting qubit. *Science* **367**, 425–428 (2020).

80. L. Bai, M. Harder, P. Hyde, Z. Zhang, C.-M. Hu, Y. P. Chen, J. Q. Xiao, Cavity mediated manipulation of distant spin currents using a cavity-magnon-polariton. *Phys. Rev. Lett.* **118**, 217201 (2017).

81. X. Zhang, C.-L. Zou, N. Zhu, F. Marquardt, L. Jiang, H. X. Tang, Magnon dark modes and gradient memory. *Nat. Commun.* **6**, 8914 (2015).

82. Z.-Q. Wang, Z.-Y. Wang, Y.-P. Wang, J. Q. You, Single-mode magnon-polariton lasing and amplification controlled by dissipative coupling. *Phys. Rev. Lett.* **135**, 186704 (2025).

83. Y.-P. Wang, C.-M. Hu, Dissipative couplings in cavity magnonics. *J. Appl. Phys.* **127**, 130901 (2020).

84. W. Song, T. Li, S. Wu, Z. Wang, C. Chen, Y. Chen, C. Huang, K. Qiu, S. Zhu, Y. Zou, T. Li, Dispersionless coupling among optical waveguides by artificial gauge field. *Phys. Rev. Lett.* **129**, 053901 (2022).

85. K. Yamaguchi, M. Nakajima, T. Suemoto, Coherent control of spin precession motion with impulsive magnetic fields of half-cycle terahertz radiation. *Phys. Rev. Lett.* **105**, 237201 (2010).

86. T. S. Seifert, S. Jaiswal, J. Barker, S. T. Weber, I. Razdolski, J. Cramer, O. Gueckstock, S. F. Maehrlein, L. Nadvornik, S. Watanabe, C. Ciccarelli, A. Melnikov, G. Jakob, M. Münzenberg, S. T. B. Goennenwein, G. Woltersdorf, B. Rethfeld, P. W. Brouwer, M. Wolf, M. Kläui, T. Kampfrath, Femtosecond formation dynamics of the spin Seebeck effect revealed by terahertz spectroscopy. *Nat. Commun.* **9**, 2899 (2018).

87. P. Lodahl, S. Mahmoodian, S. Stobbe, Interfacing single photons and single quantum dots with photonic nanostructures. *Rev. Mod. Phys.* **87**, 347–400 (2015).

88. M. Arcari, I. Söllner, A. Javadi, S. Lindskov Hansen, S. Mahmoodian, J. Liu, H. Thyrrestrup, E. H. Lee, J. D. Song, S. Stobbe, P. Lodahl, Near-unity coupling efficiency of a quantum emitter to a photonic crystal waveguide. *Phys. Rev. Lett.* **113**, 093603 (2014).

89. J.-T. Shen, S. Fan, Theory of single-photon transport in a single-mode waveguide. I. Coupling to a cavity containing a two-level atom. *Phys. Rev. A* **79**, 023837 (2009).

90. W. Nie, T. Shi, F. Nori, Y.-X. Liu, Topology-enhanced nonreciprocal scattering and photon absorption in a waveguide. *Phys. Rev. Appl.* **15**, 044041 (2021).
